# Supplementary material for: Phytochemicals from Pterocarpus angolensis DC and Their Cytotoxic Activities against Breast Cancer Cells
Source: Plants (Basel). 2024 Jan 19;13(2):301. doi: 10.3390/plants13020301 (PMC10818308; doi:10.3390/plants13020301)
Supplement: Supplementary file 1 [file plants-13-00301-s001.zip › plants-2784620-supplementary.pdf]

Legends for Figures

Figure S1: ^{1}H NMR spectrum of friedelan-3-one (**1**) recorded in $CDCl_3$.

Figure S2: ^{13}C NMR spectrum of friedelan-3-one (**1**) recorded in $CDCl_3$.

Figure S3: MS spectrum of friedelan-3-one (**1**).

Figure S4: FT-IR spectrum of friedelan-3-one (**1**)

Figure S5: ^{1}H NMR spectrum of 3α -Hydroxyfriedel-2-one (**2**) recorded in $CDCl_3$.

Figure S6: ^{13}C NMR spectrum of 3α -Hydroxyfriedel-2-one (**2**)recorded in $CDCl_3$.

Figure S7: MS spectrum of 3α -Hydroxyfriedel-2-one (**2**).

Figure S8: FT-IR spectrum of 3α -Hydroxyfriedel-2-one (**2**).

Figure S9: ^{1}H NMR spectrum of 3-Hydroxyfriedel-3-en-2-one (**3**) recorded in $CDCl_3$.

Figure S10: ^{13}C NMR spectrum of 3-Hydroxyfriedel-3-en-2-one (**3**) recorded in $CDCl_3$.

Figure S11: MS spectrum of 3-Hydroxyfriedel-3-en-2-one (**3**).

Figure S12: FT-IR spectrum of 3-Hydroxyfriedel-3-en-2-one (**3**).

Figure S13: ^{1}H NMR spectrum of Lupeol (**4**) recorded in $CDCl_3$.

Figure S14: ^{13}C NMR spectrum of Lupeol (**4**) recorded in $CDCl_3$.

Figure S15: MS spectrum of Lupeol (**4**).

Figure S16: FT-IR spectrum of Lupeol (**4**).

Figure S17: ^{1}H NMR spectrum of Stigmasterol (**5**) recorded in $CDCl_3$.

Figure S18: ^{13}C NMR spectrum of Stigmasterol (**5**) recorded in $CDCl_3$

Figure S19: MS spectrum of Stigmasterol (**5**).

Figure S20: FT-IR spectrum of Stigmasterol (**5**).

Figure S21: ^{1}H NMR spectrum of (±) -4-*O*-Methylangolensin (**6**) recorded in $CDCl_3$.

Figure S22: ^{13}C NMR spectrum of (±) -4-*O*-Methylangolensin (**6**) recorded in $CDCl_3$

Figure S23: MS spectrum of (±) -4-*O*-Methylangolensin (**6**).

Figure S24: FT-IR spectrum of (±) -4-*O*-Methylangolensin (**6**).

Figure S25. ^{1}H NMR spectrum of Oleanolic acid acetate (**7**) recorded in $CDCl_3$.

Figure S26: ^{13}C NMR spectrum of Oleanolic acid acetate (**7**) recorded in $CDCl_3$

Figure S27: MS spectrum of Oleanolic acid acetate (**7**).

Figure S28: FT-IR spectrum of Oleanolic acid acetate (**7**).

Figure S29: ^{1}H NMR spectrum of Tetradecyl (E)-ferulate (**8**) recorded in $CDCl_3$.

Figure S30: ^{13}C NMR spectrum of Tetradecyl (E)-ferulate (**8**) recorded in $CDCl_3$

Figure S31: MS spectrum of Tetradecyl (E)-ferulate (**8**).

Figure S32: FT-IR spectrum of Tetradecyl (E)-ferulate (**8**).

Figure S33: ^{1}H NMR spectrum of 9-Octadecenoic acid (**9**) recorded in $CDCl_3$.

Figure S34: ^{13}C NMR spectrum of 9-Octadecenoic acid (**9**) recorded in $CDCl_3$

Figure S35: MS spectrum of 9-Octadecenoic acid (**9**).

Figure S36: FT-IR spectrum of 9-Octadecenoic acid (**9**).

Table S1: ^{13}C NMR (100 MHz) data (δ value) of isolated compounds (1-8) in $CHCl_3$

Table S2: ^{1}H NMR (400 MHz) data (δ value) of isolated compounds (1-5 and 7) in $CHCl_3$

Table S3: ^{1}H NMR (400 MHz) data (δ value) of isolated compounds (6 and 8) in CHCl

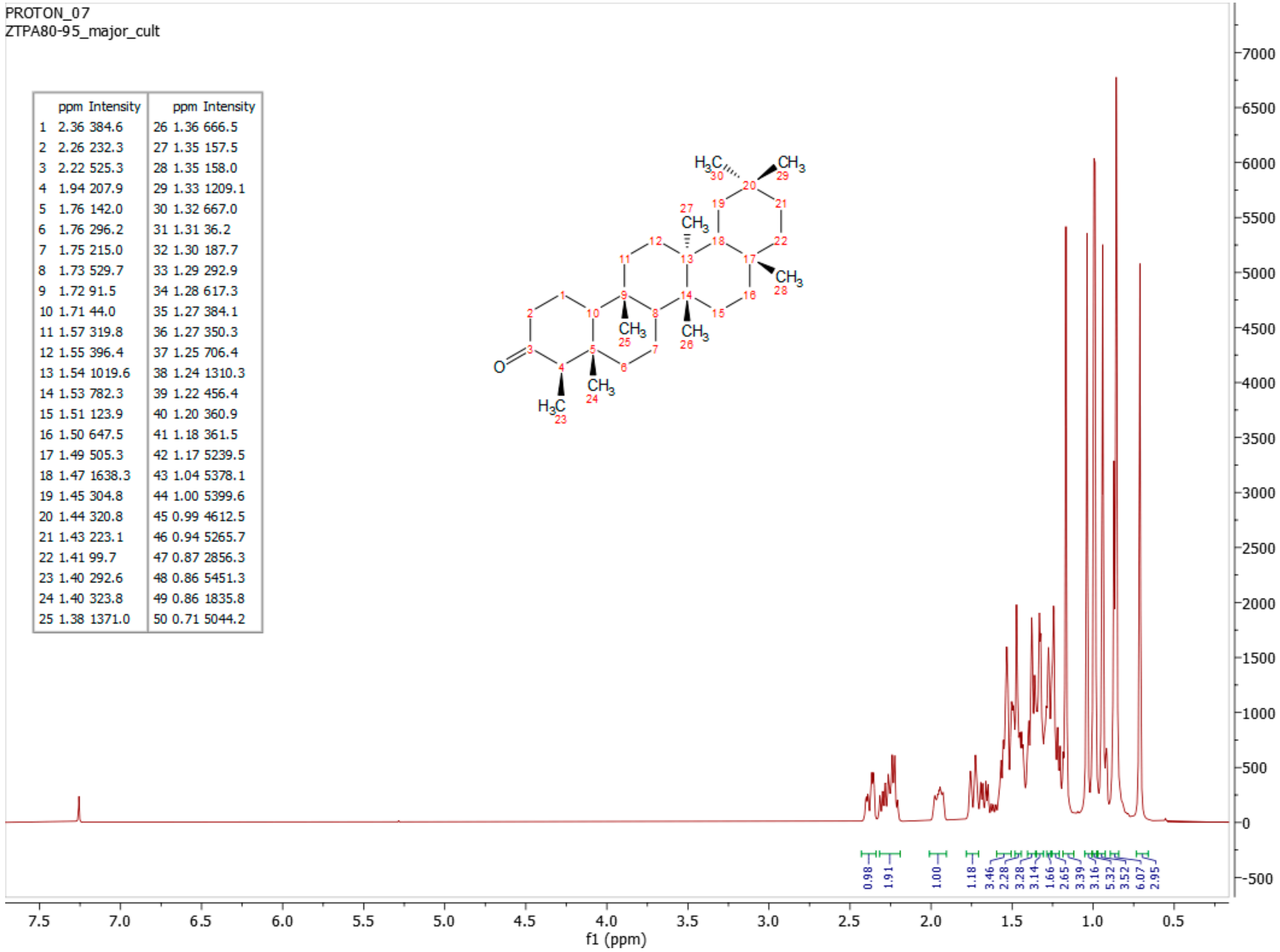

Figure SI: ^{1}H NMR spectrum of friedelan-3-one (**1**) recorded in $CDCl_3$.

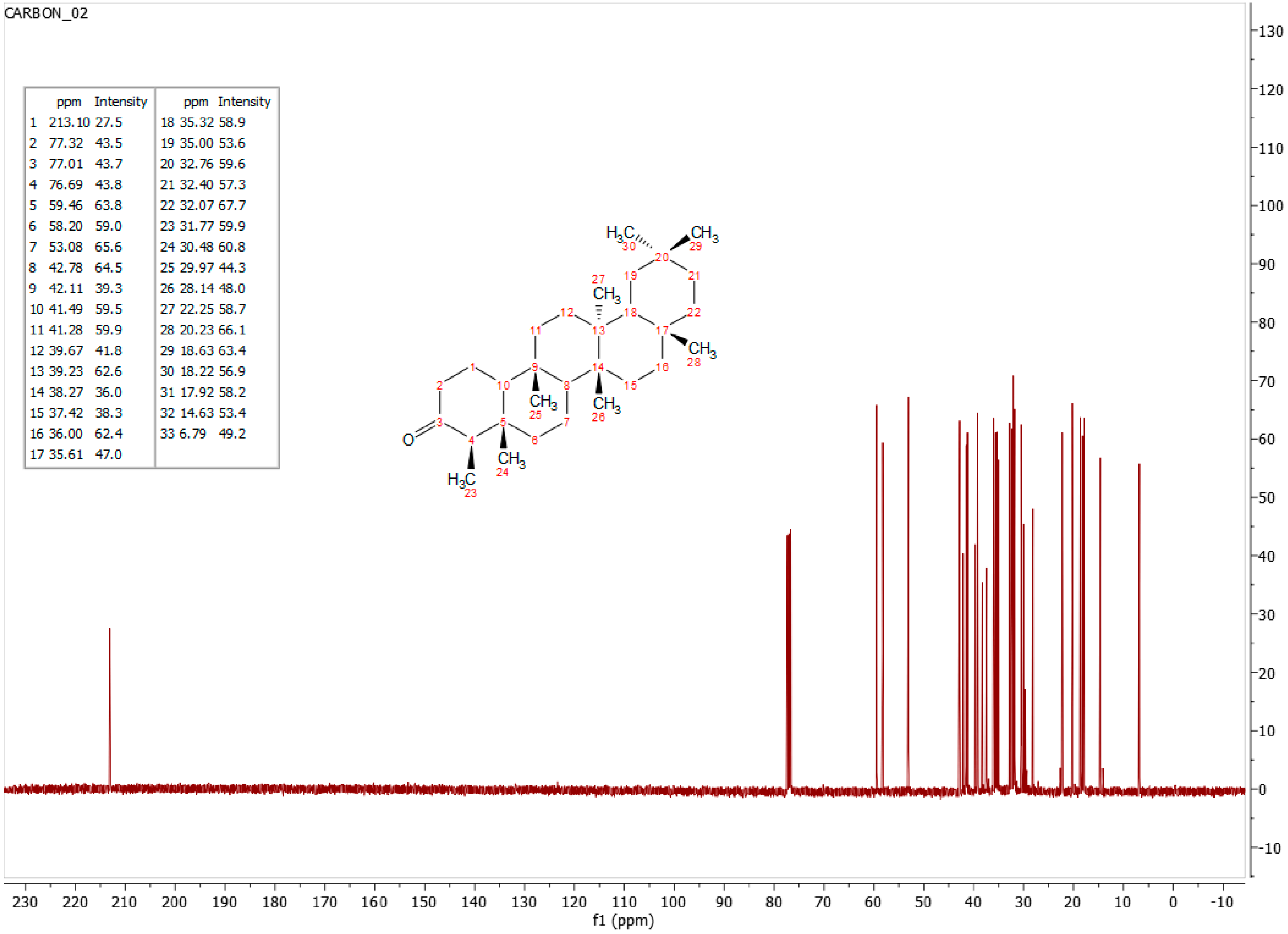

Figure S2: ^{13}C NMR spectrum of friedelan-3-one (**1**) recorded in $CDCl_3$.

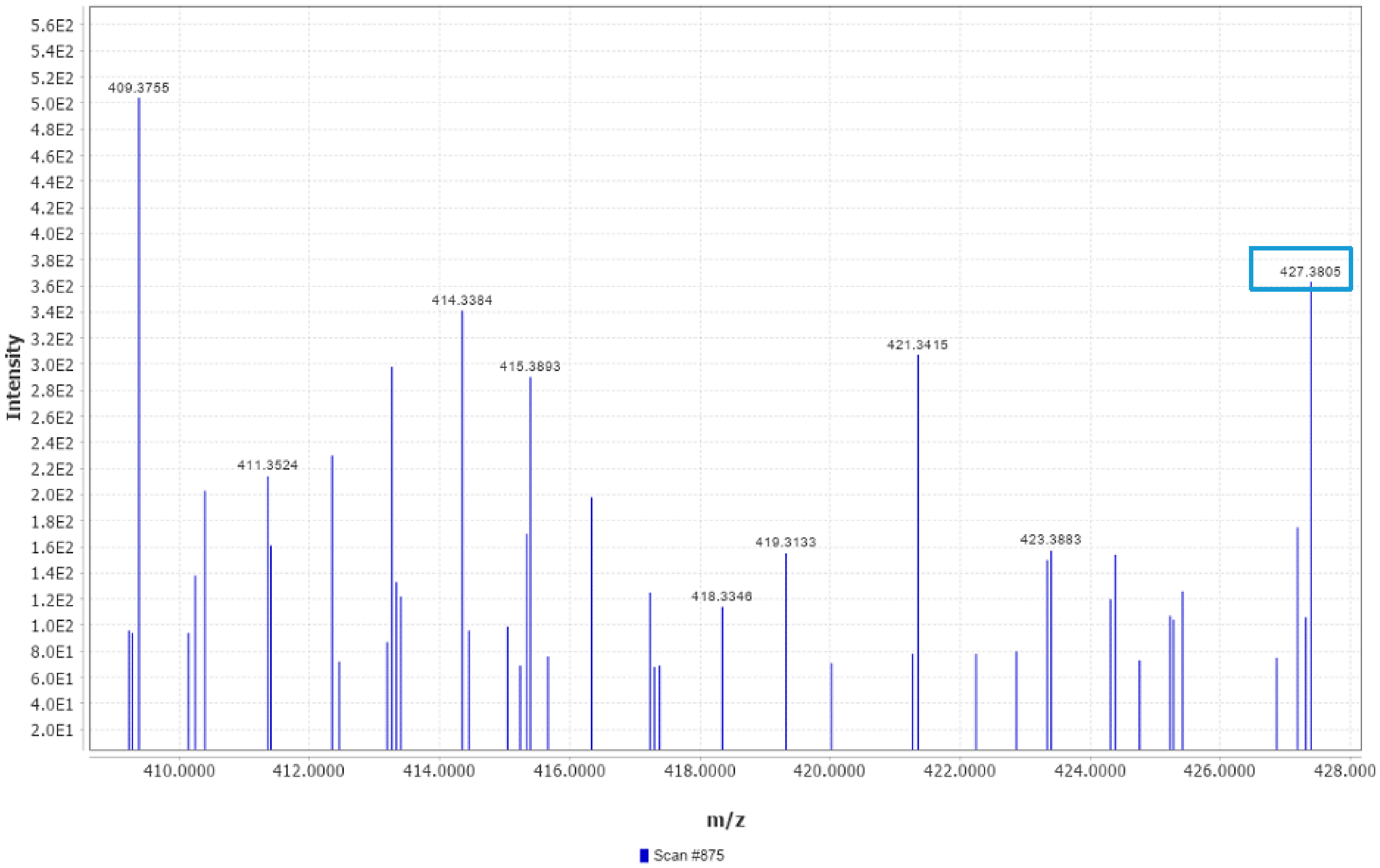

Figure S3: MS spectrum of friedelan-3-one (**1**).

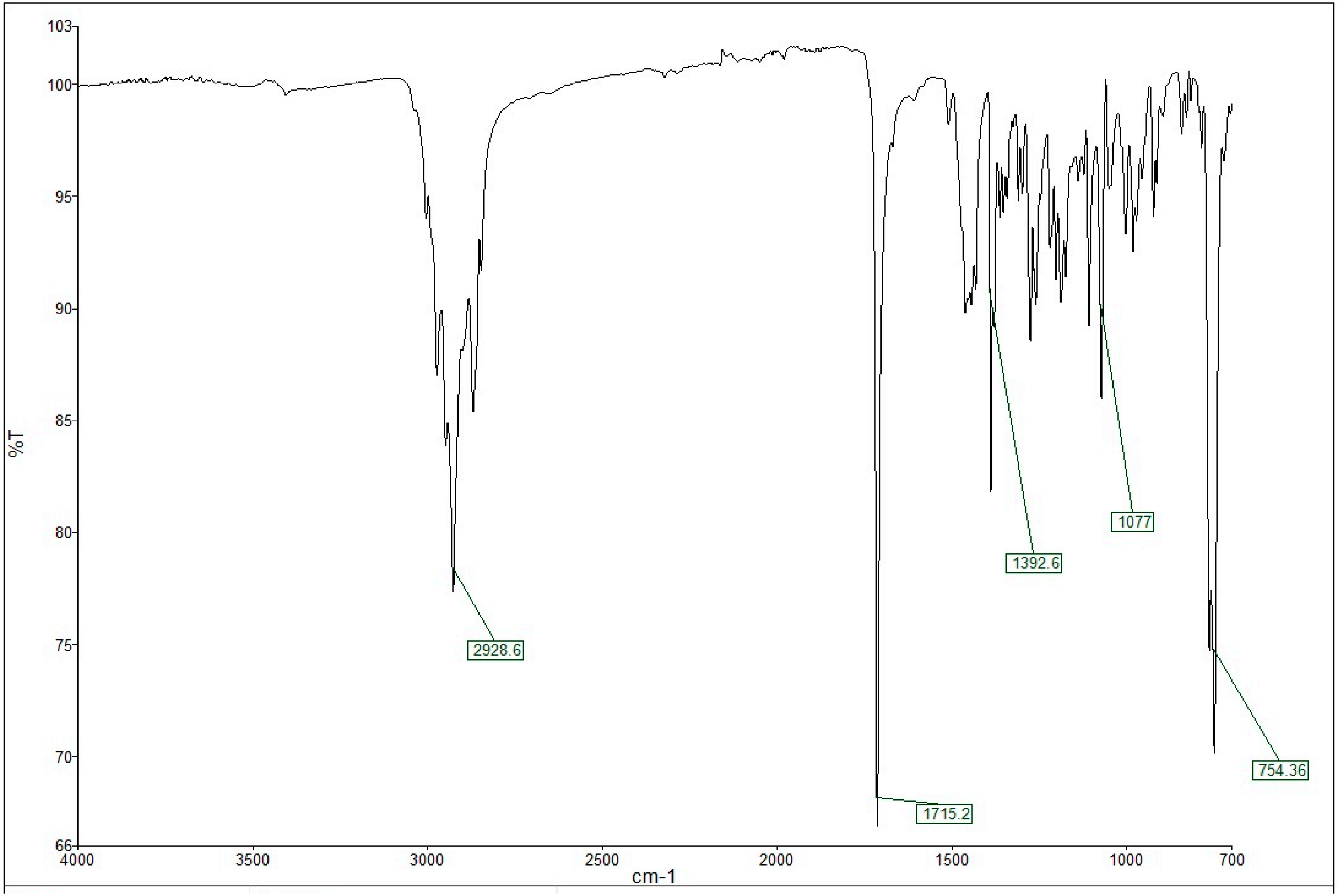

Figure S4: FT-IR spectrum of friedelan-3-one (**1**).

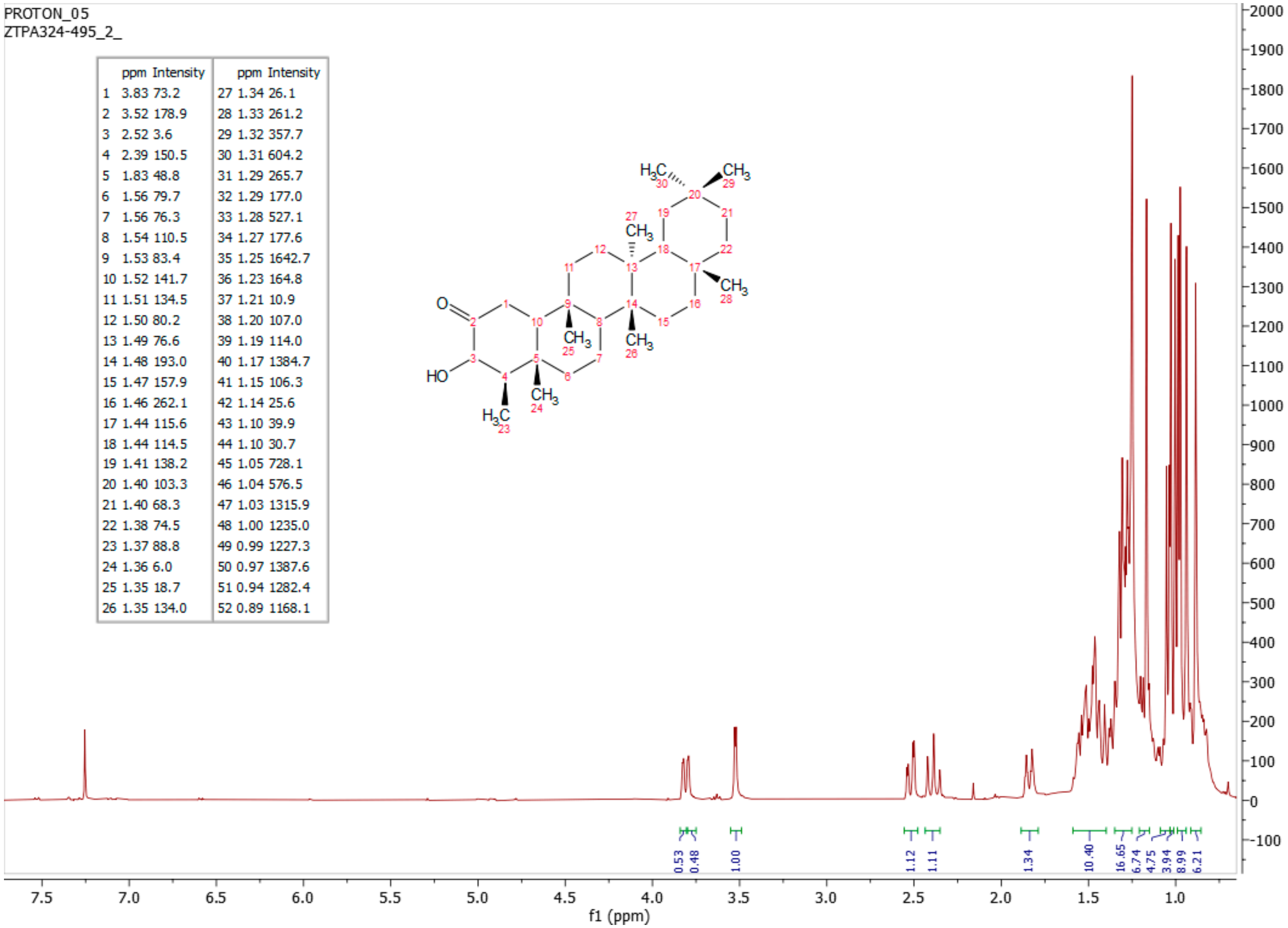

Figure S5: ^{1}H NMR spectrum of 3α -Hydroxyfriedel-2-one (**2**) recorded in $CDCl_3$.

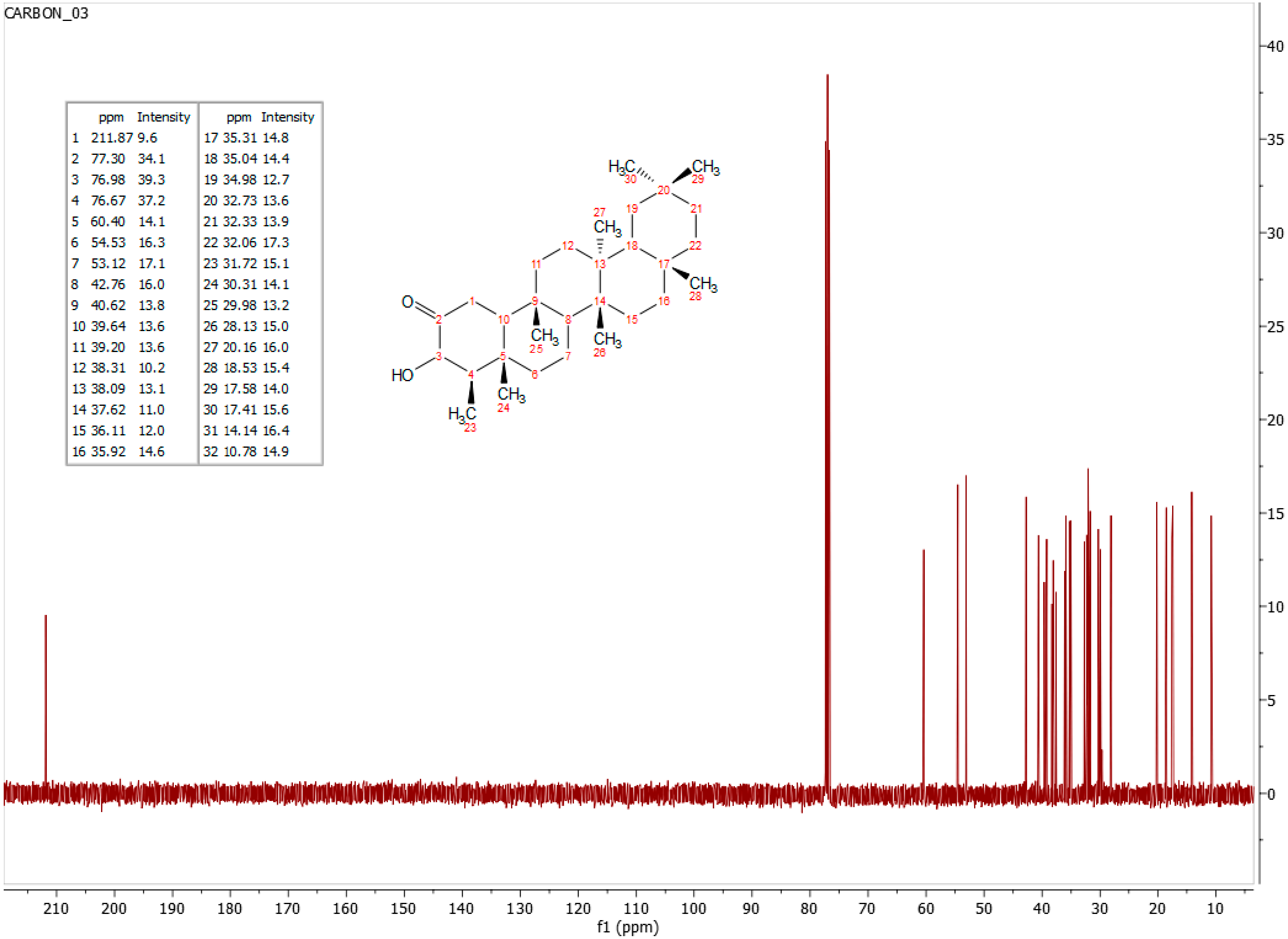

	ppm	Intensity		ppm	Intensity
1	211.87	9.6	17	35.31	14.8
2	77.30	34.1	18	35.04	14.4
3	76.98	39.3	19	34.98	12.7
4	76.67	37.2	20	32.73	13.6
5	60.40	14.1	21	32.33	13.9
6	54.53	16.3	22	32.06	17.3
7	53.12	17.1	23	31.72	15.1
8	42.76	16.0	24	30.31	14.1
9	40.62	13.8	25	29.98	13.2
10	39.64	13.6	26	28.13	15.0
11	39.20	13.6	27	20.16	16.0
12	38.31	10.2	28	18.53	15.4
13	38.09	13.1	29	17.58	14.0
14	37.62	11.0	30	17.41	15.6
15	36.11	12.0	31	14.14	16.4
16	35.92	14.6	32	10.78	14.9

Figure S6: ^{13}C NMR spectrum of 3α -Hydroxyfriedel-2-one (**2**) recorded in $CDCl_3$.

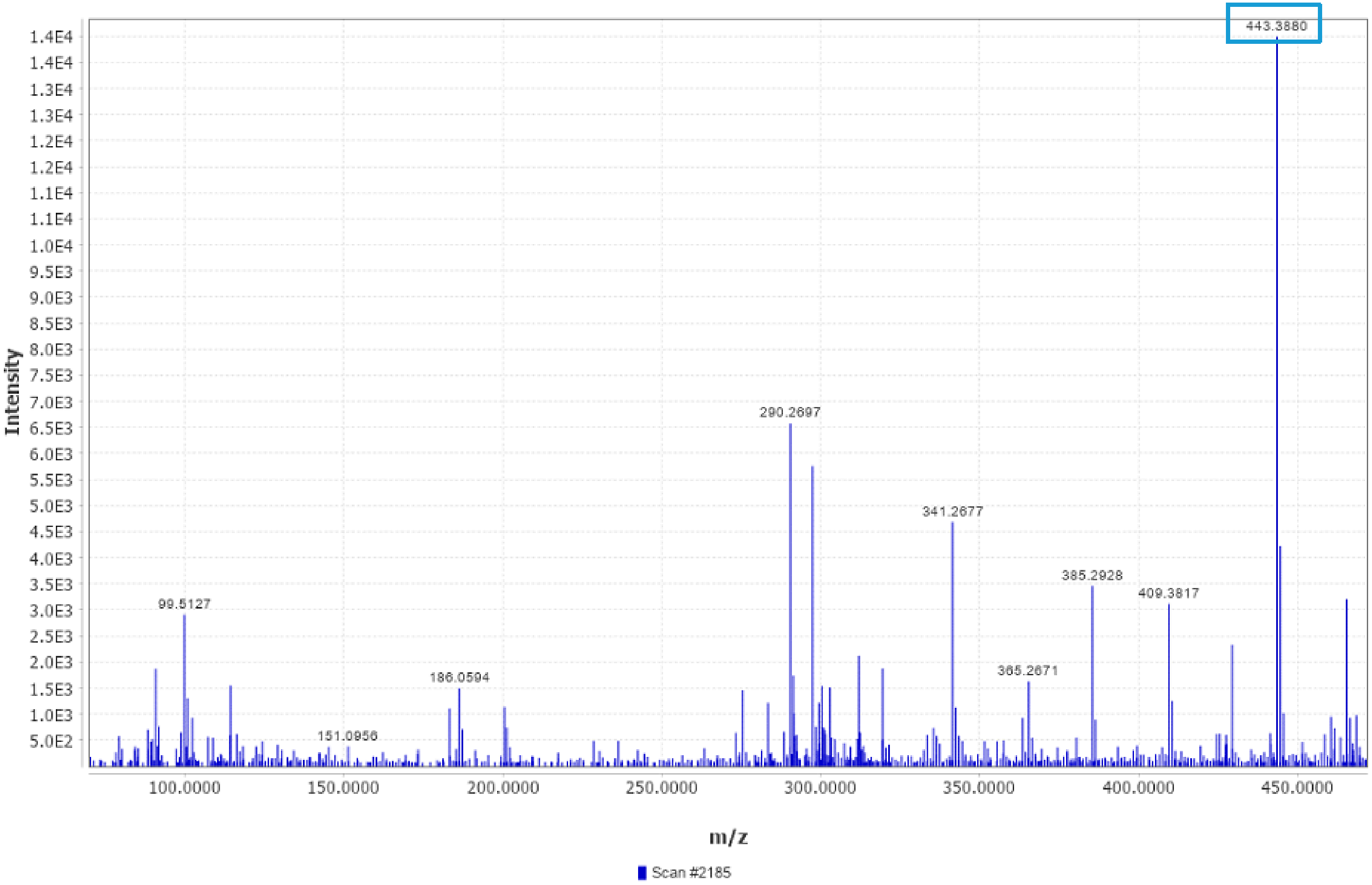

Figure S7: MS spectrum of 3α -Hydroxyfriedel-2-one (**2**).

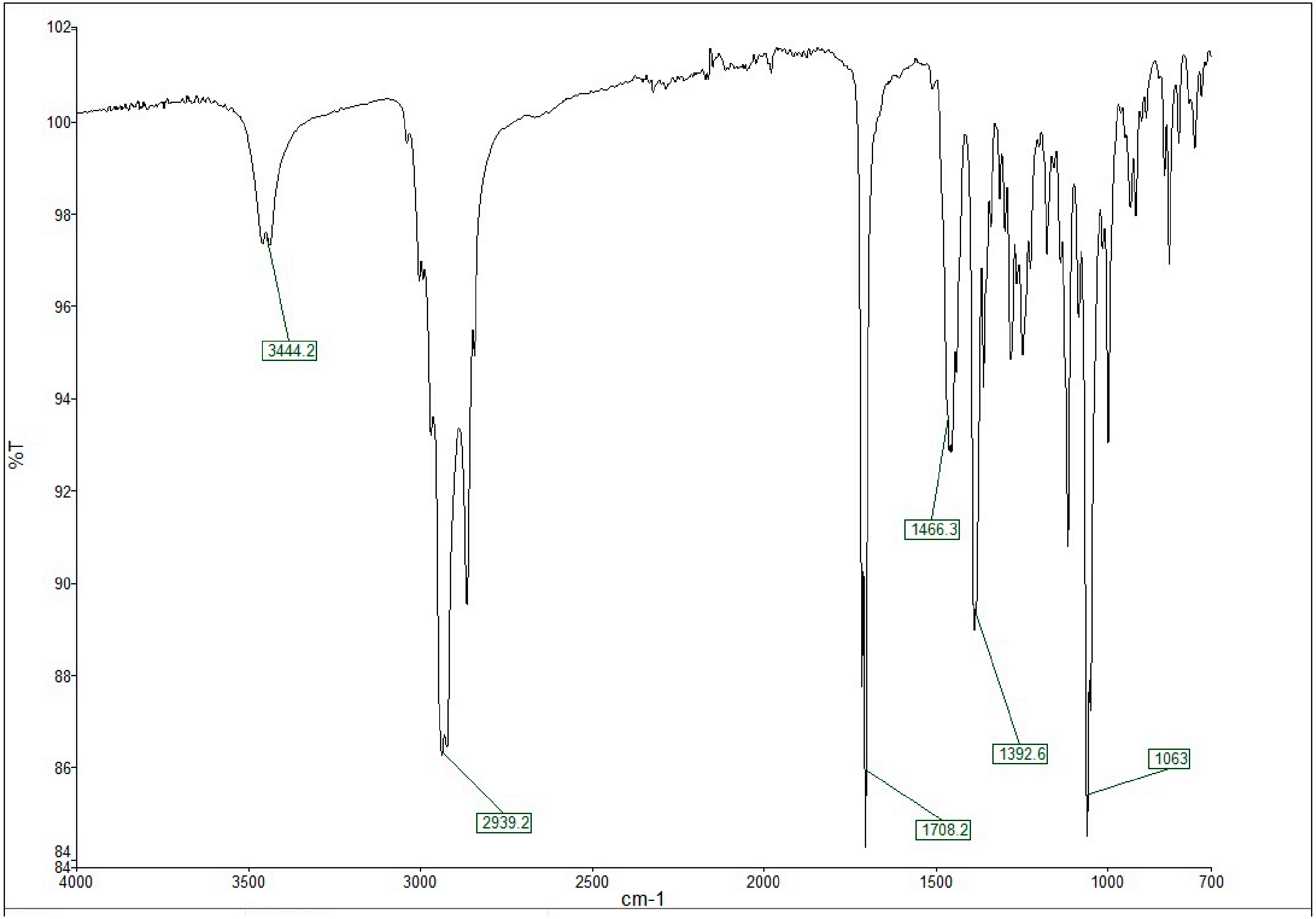

Figure S8: FT-IR spectrum of 3α -Hydroxyfriedel-2-one (**2**).

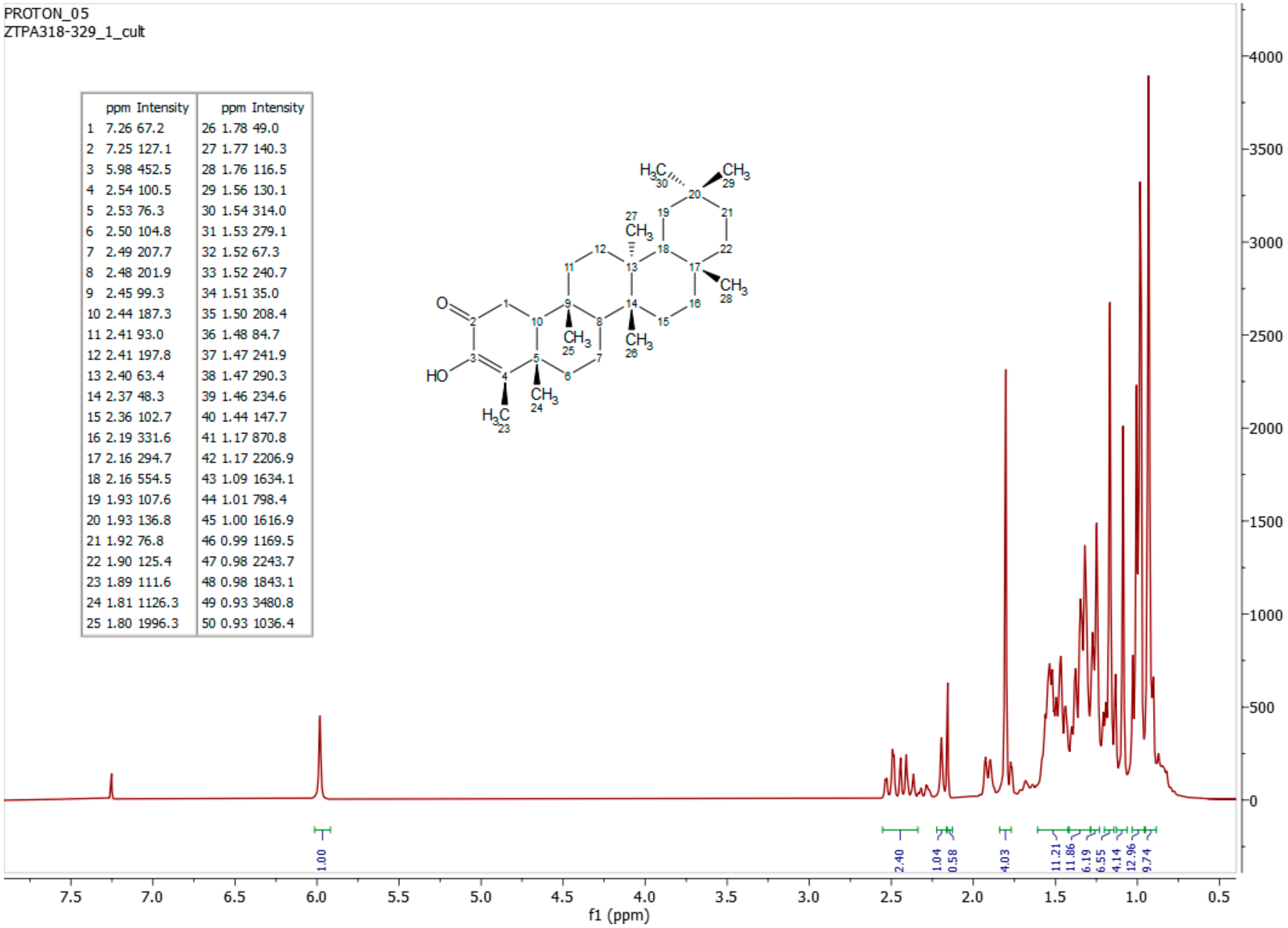

	ppm	Intensity		ppm	Intensity
1	7.26	67.2	26	1.78	49.0
2	7.25	127.1	27	1.77	140.3
3	5.98	452.5	28	1.76	116.5
4	2.54	100.5	29	1.56	130.1
5	2.53	76.3	30	1.54	314.0
6	2.50	104.8	31	1.53	279.1
7	2.49	207.7	32	1.52	67.3
8	2.48	201.9	33	1.52	240.7
9	2.45	99.3	34	1.51	35.0
10	2.44	187.3	35	1.50	208.4
11	2.41	93.0	36	1.48	84.7
12	2.41	197.8	37	1.47	241.9
13	2.40	63.4	38	1.47	290.3
14	2.37	48.3	39	1.46	234.6
15	2.36	102.7	40	1.44	147.7
16	2.19	331.6	41	1.17	870.8
17	2.16	294.7	42	1.17	2206.9
18	2.16	554.5	43	1.09	1634.1
19	1.93	107.6	44	1.01	798.4
20	1.93	136.8	45	1.00	1616.9
21	1.92	76.8	46	0.99	1169.5
22	1.90	125.4	47	0.98	2243.7
23	1.89	111.6	48	0.98	1843.1
24	1.81	1126.3	49	0.93	3480.8
25	1.80	1996.3	50	0.93	1036.4

Figure S9: ^{1}H NMR spectrum of 3-Hydroxyfriedel-3-en-2-one (**3**) recorded in $CDCl_3$.

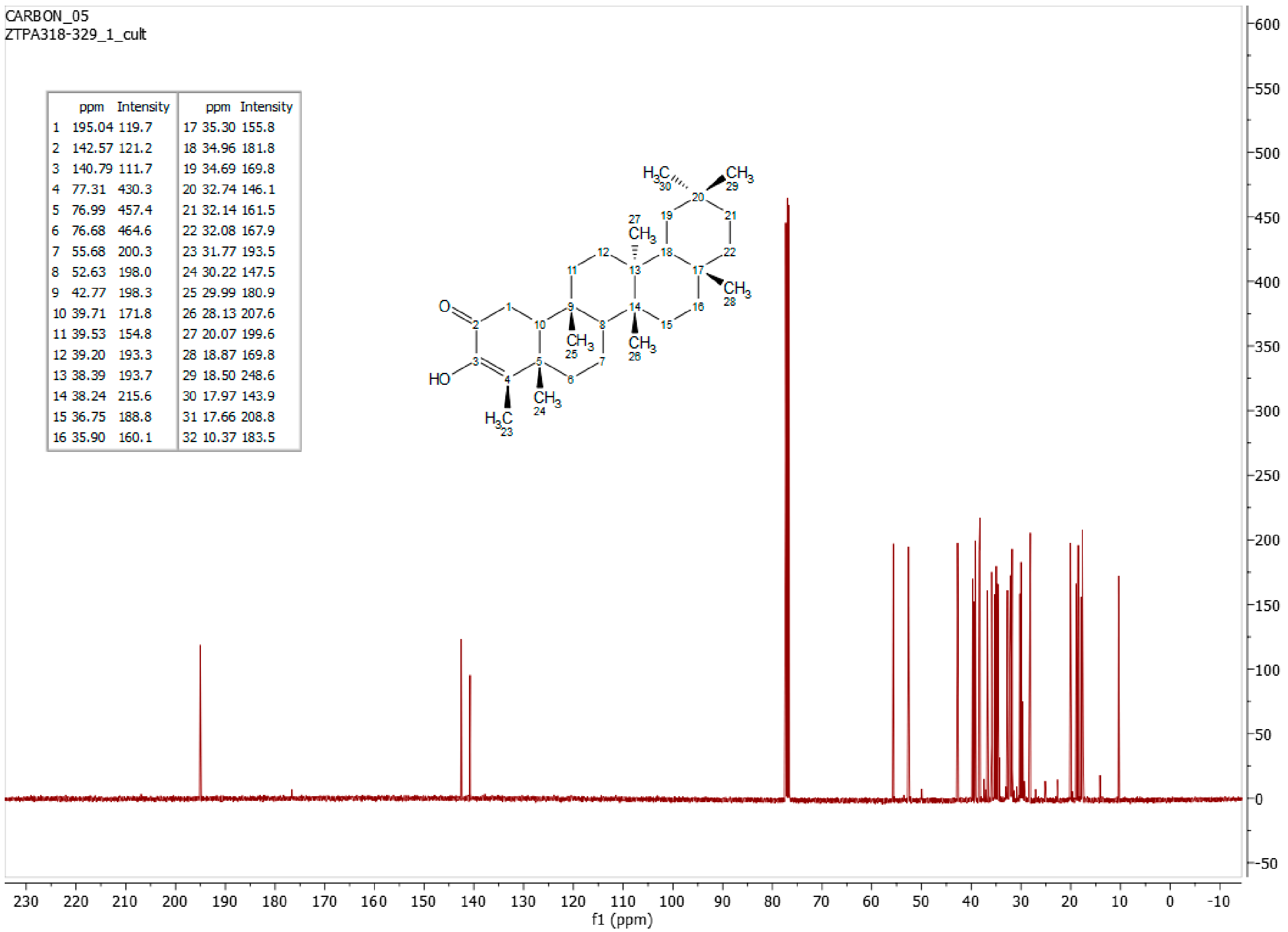

	ppm	Intensity		ppm	Intensity
1	195.04	119.7	17	35.30	155.8
2	142.57	121.2	18	34.96	181.8
3	140.79	111.7	19	34.69	169.8
4	77.31	430.3	20	32.74	146.1
5	76.99	457.4	21	32.14	161.5
6	76.68	464.6	22	32.08	167.9
7	55.68	200.3	23	31.77	193.5
8	52.63	198.0	24	30.22	147.5
9	42.77	198.3	25	29.99	180.9
10	39.71	171.8	26	28.13	207.6
11	39.53	154.8	27	20.07	199.6
12	39.20	193.3	28	18.87	169.8
13	38.39	193.7	29	18.50	248.6
14	38.24	215.6	30	17.97	143.9
15	36.75	188.8	31	17.66	208.8
16	35.90	160.1	32	10.37	183.5

Figure S10: ^{13}C NMR spectrum of 3-Hydroxyfriedel-3-en-2-one (**3**) recorded in $CDCl_3$.

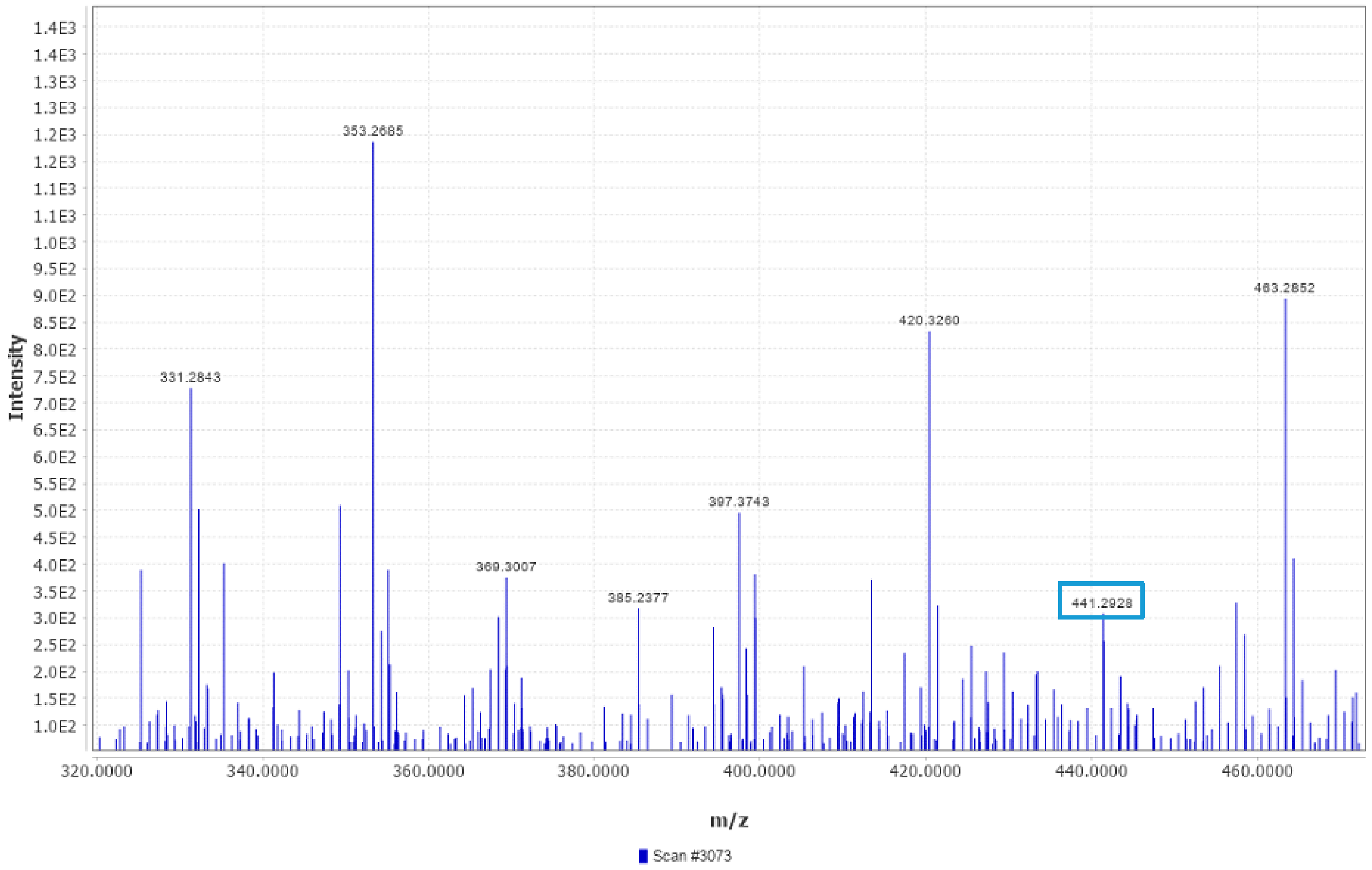

Figure S11: MS spectrum of 3-Hydroxyfriedel-3-en-2-one (**3**).

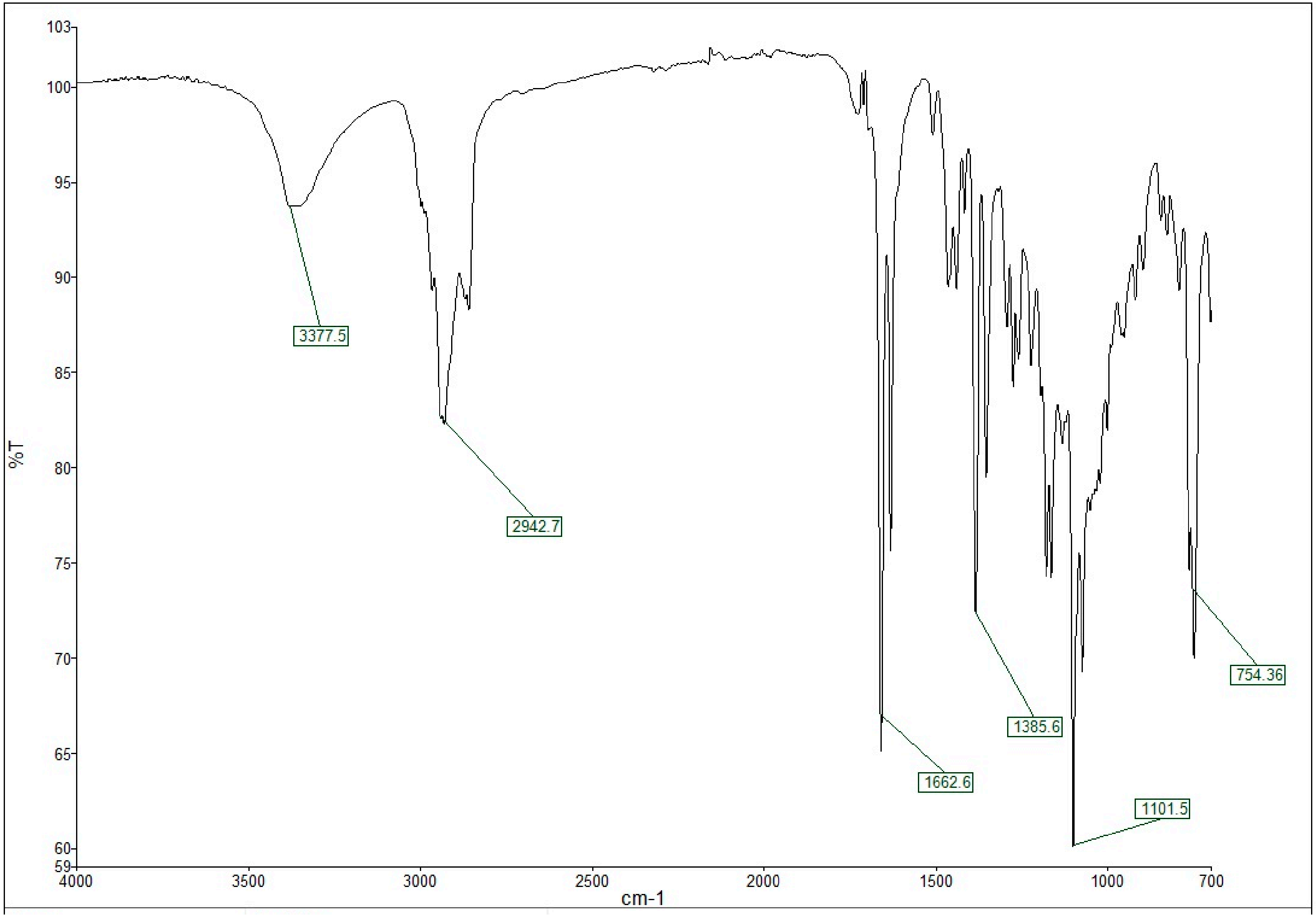

Figure S12: FT-IR spectrum of 3-Hydroxyfriedel-3-en-2-one (**3**)

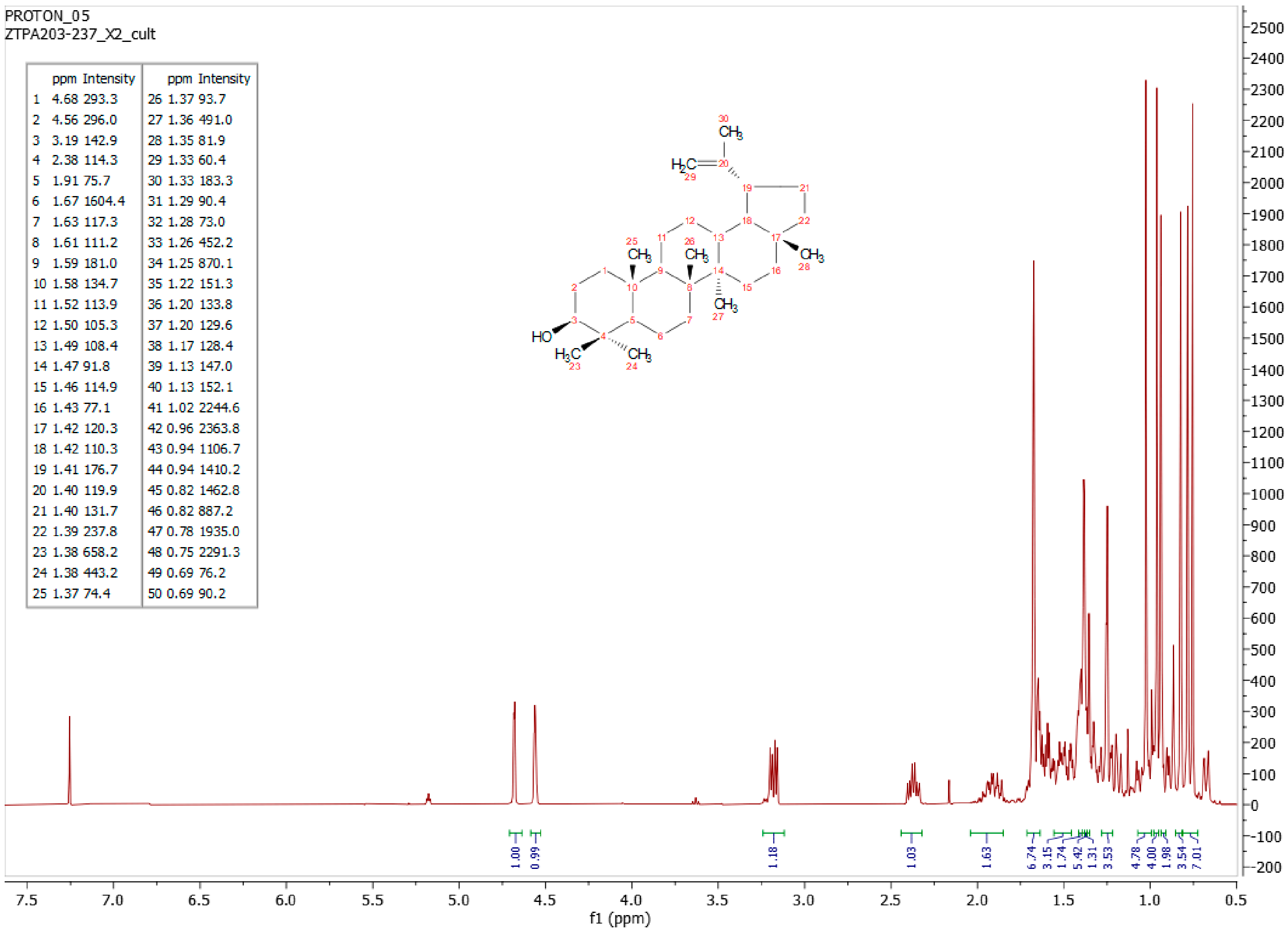

	ppm	Intensity		ppm	Intensity
1	4.68	293.3	26	1.37	93.7
2	4.56	296.0	27	1.36	491.0
3	3.19	142.9	28	1.35	81.9
4	2.38	114.3	29	1.33	60.4
5	1.91	75.7	30	1.33	183.3
6	1.67	1604.4	31	1.29	90.4
7	1.63	117.3	32	1.28	73.0
8	1.61	111.2	33	1.26	452.2
9	1.59	181.0	34	1.25	870.1
10	1.58	134.7	35	1.22	151.3
11	1.52	113.9	36	1.20	133.8
12	1.50	105.3	37	1.20	129.6
13	1.49	108.4	38	1.17	128.4
14	1.47	91.8	39	1.13	147.0
15	1.46	114.9	40	1.13	152.1
16	1.43	77.1	41	1.02	2244.6
17	1.42	120.3	42	0.96	2363.8
18	1.42	110.3	43	0.94	1106.7
19	1.41	176.7	44	0.94	1410.2
20	1.40	119.9	45	0.82	1462.8
21	1.40	131.7	46	0.82	887.2
22	1.39	237.8	47	0.78	1935.0
23	1.38	658.2	48	0.75	2291.3
24	1.38	443.2	49	0.69	76.2
25	1.37	74.4	50	0.69	90.2

Figure S13: ^{1}H NMR spectrum of lupeol (**4**) recorded in $CDCl_3$.

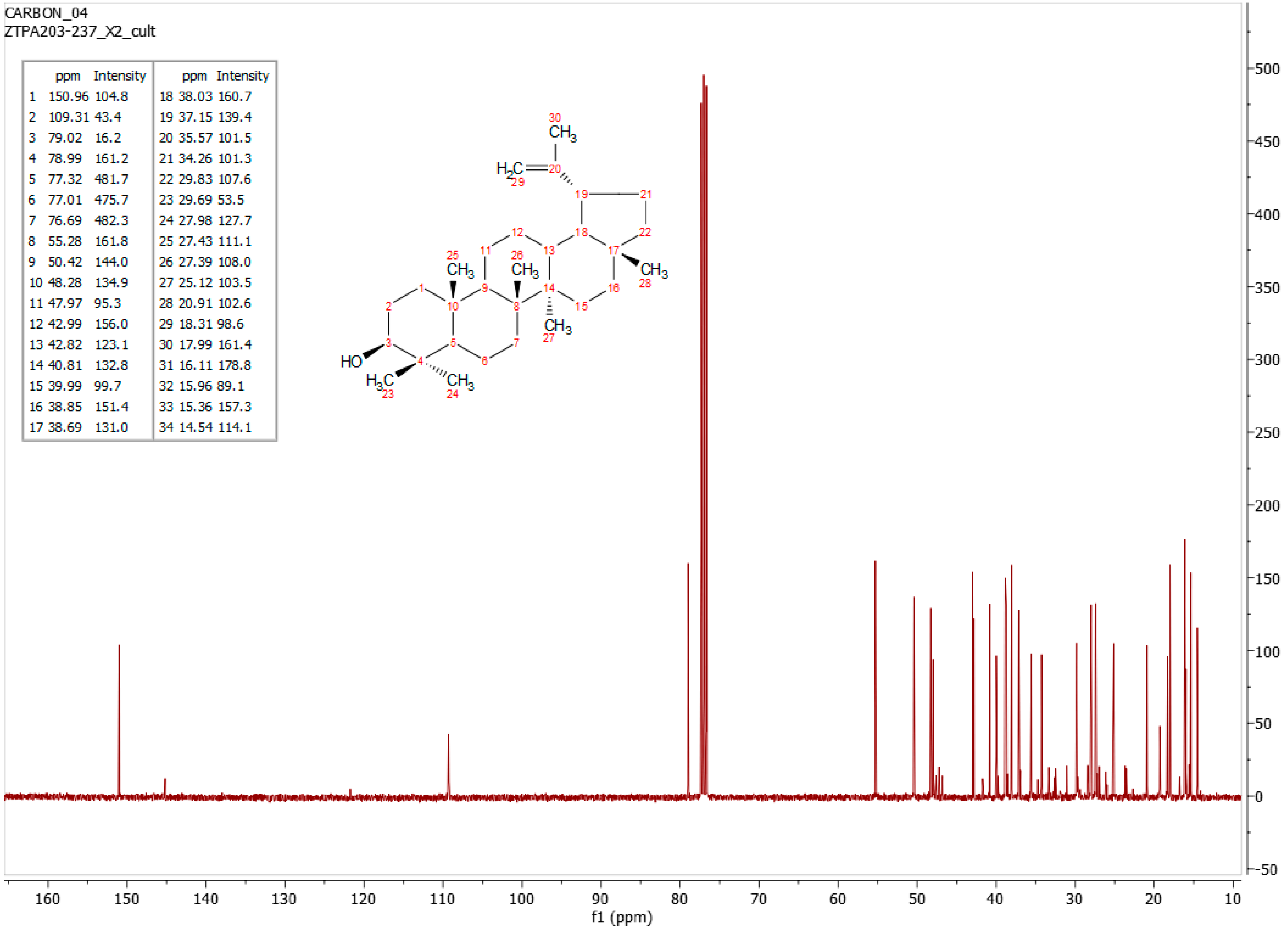

Figure S14: ^{13}C NMR spectrum of lupeol (**4**) recorded in $CDCl_3$.

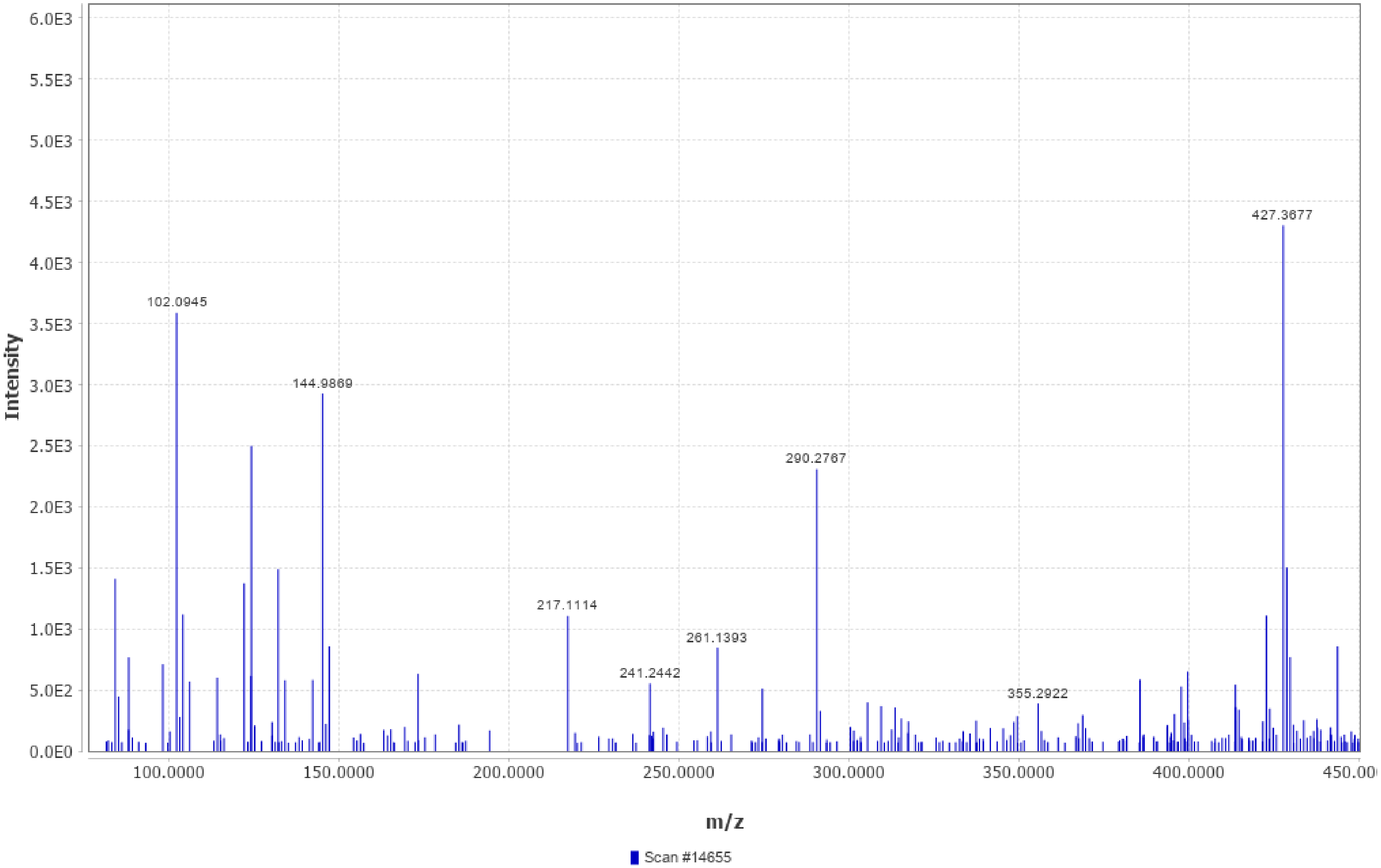

Figure S15: MS spectrum of lupeol (**4**).

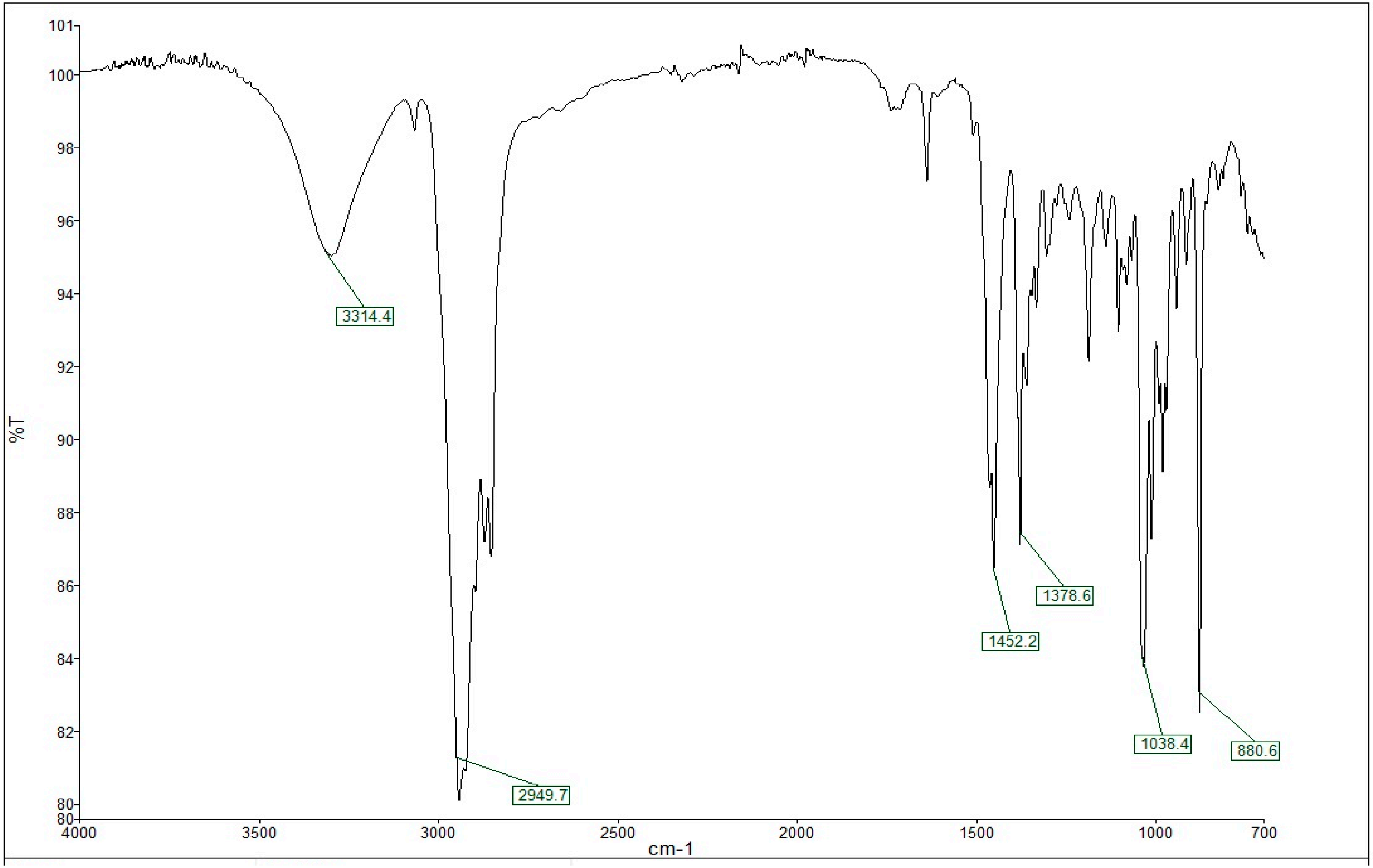

Figure S16: FT-IR spectrum of lupeol (**4**)

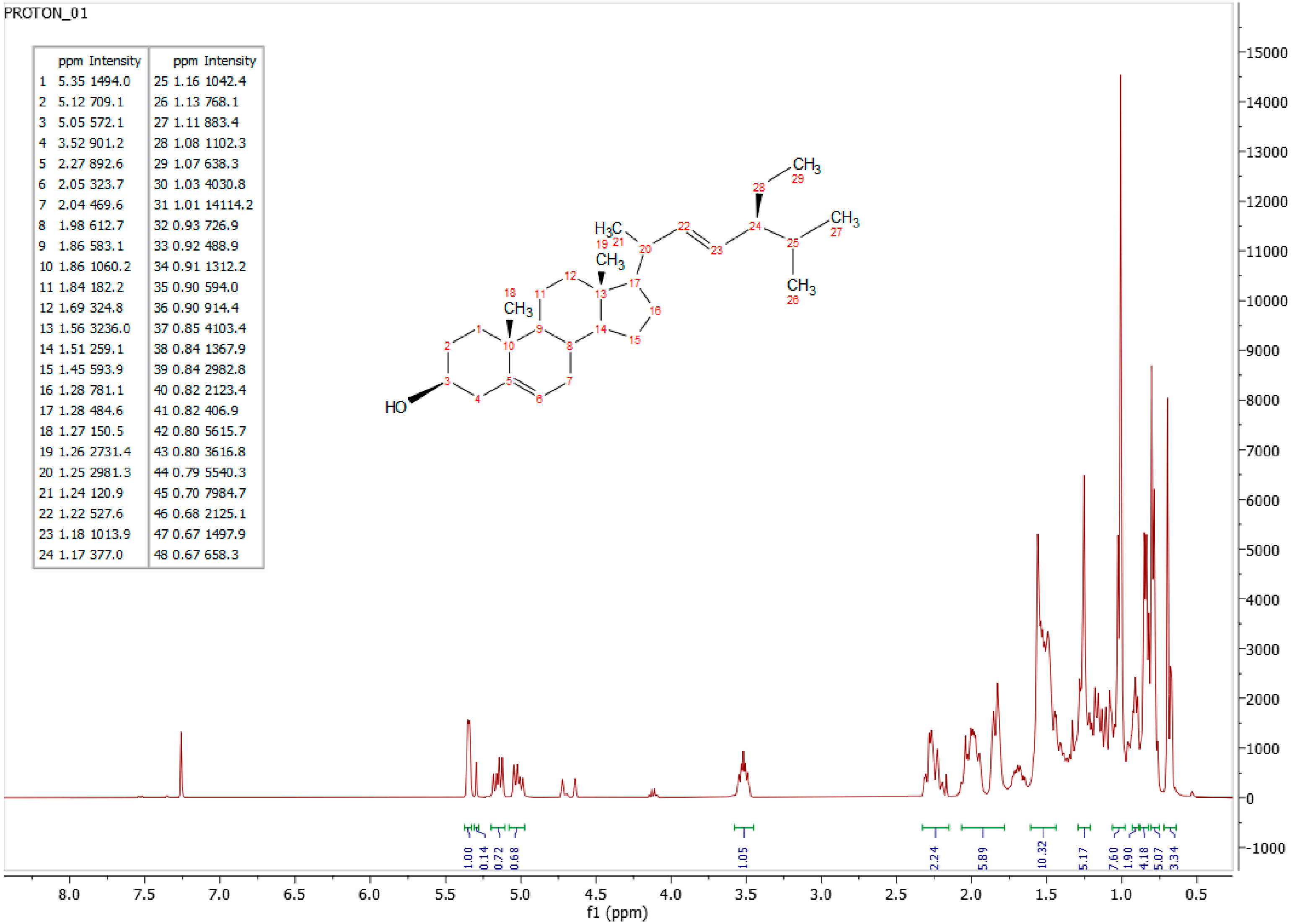

Figure S17: ^{1}H NMR spectrum of stigmasterol (**5**) recorded in $CDCl_3$.

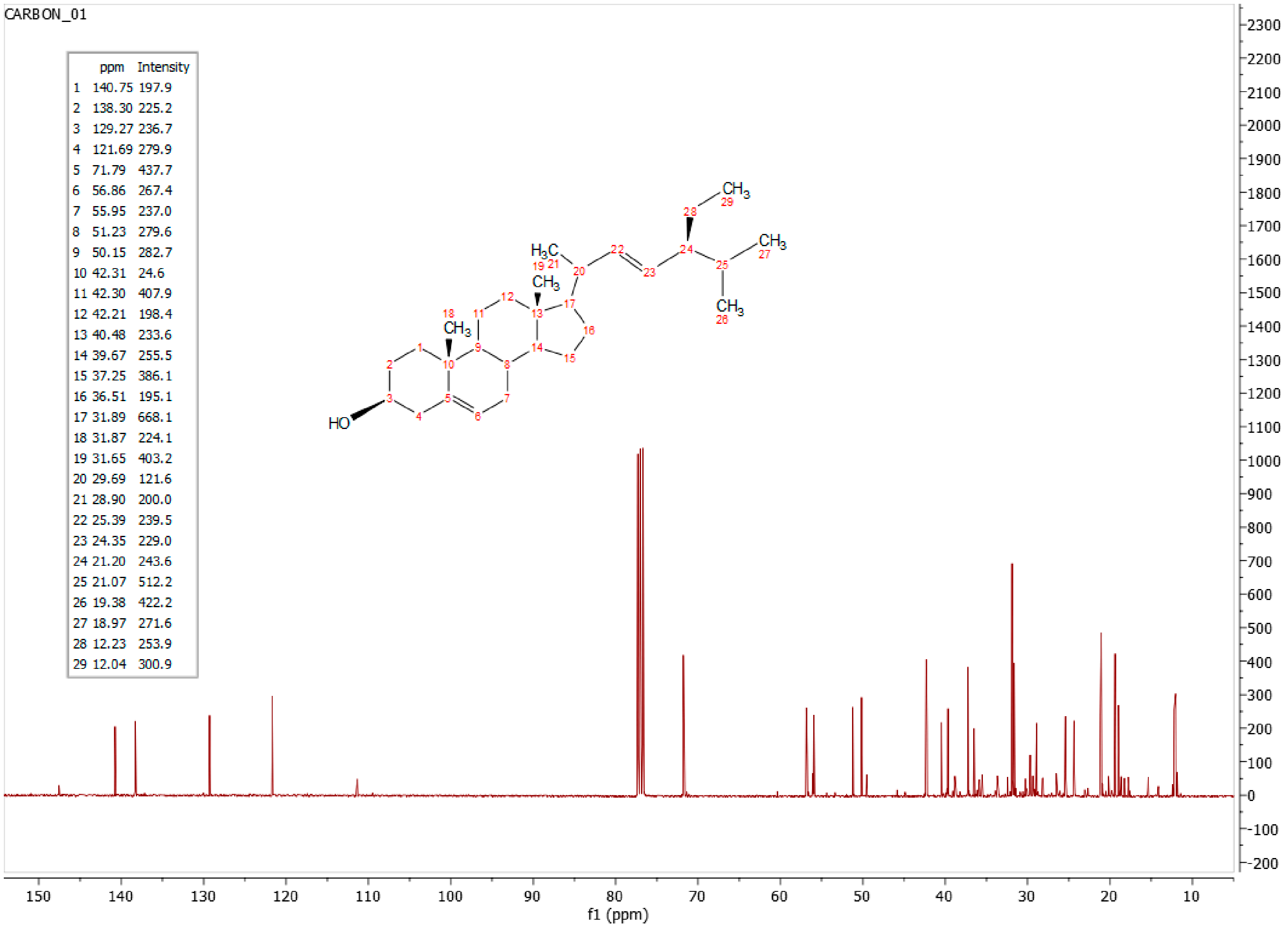

Figure S18: ^{13}C NMR spectrum of stigmasterol (**5**) recorded in $CDCl_3$

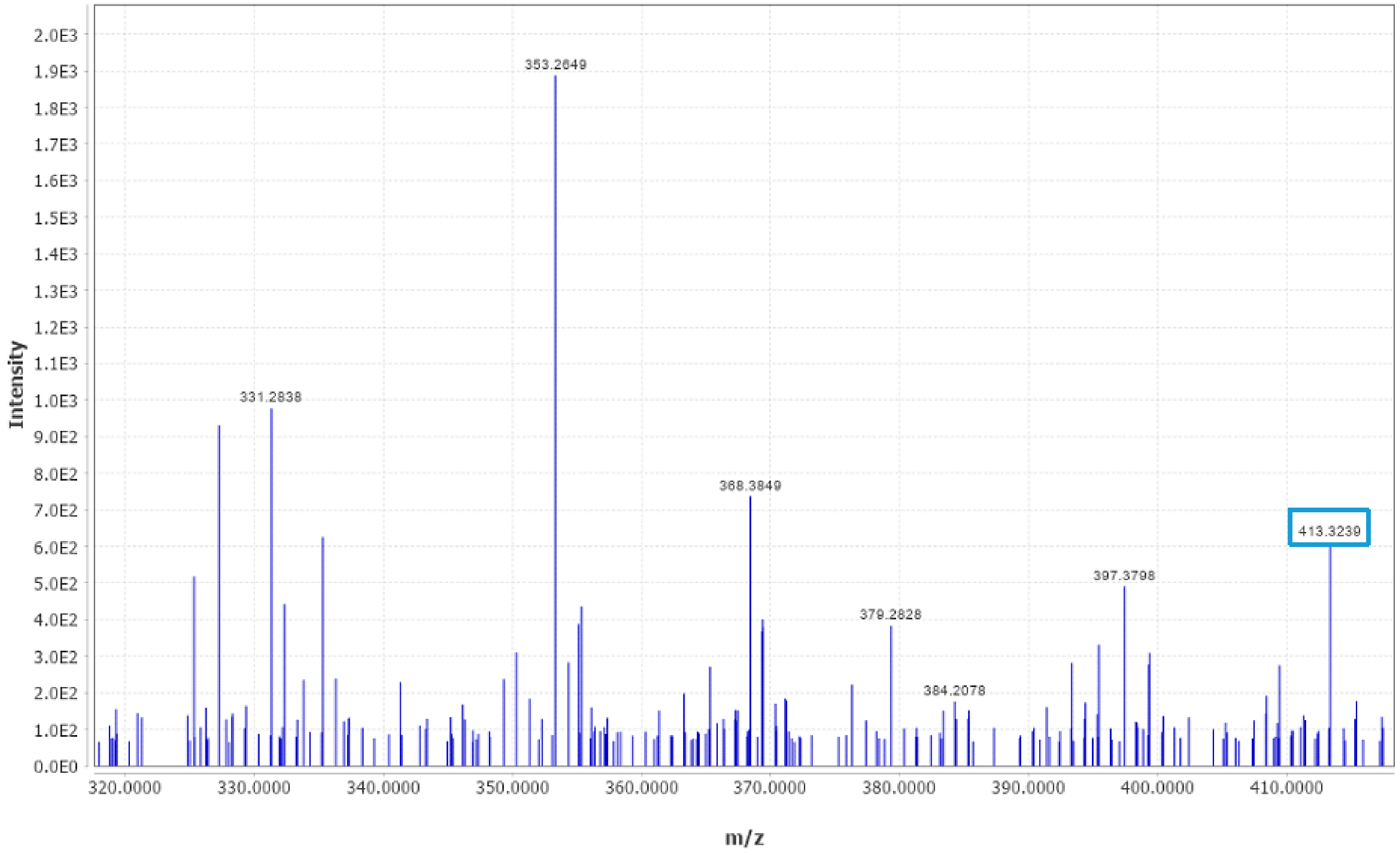

Figure S19: MS spectrum of stigmasterol (**5**).

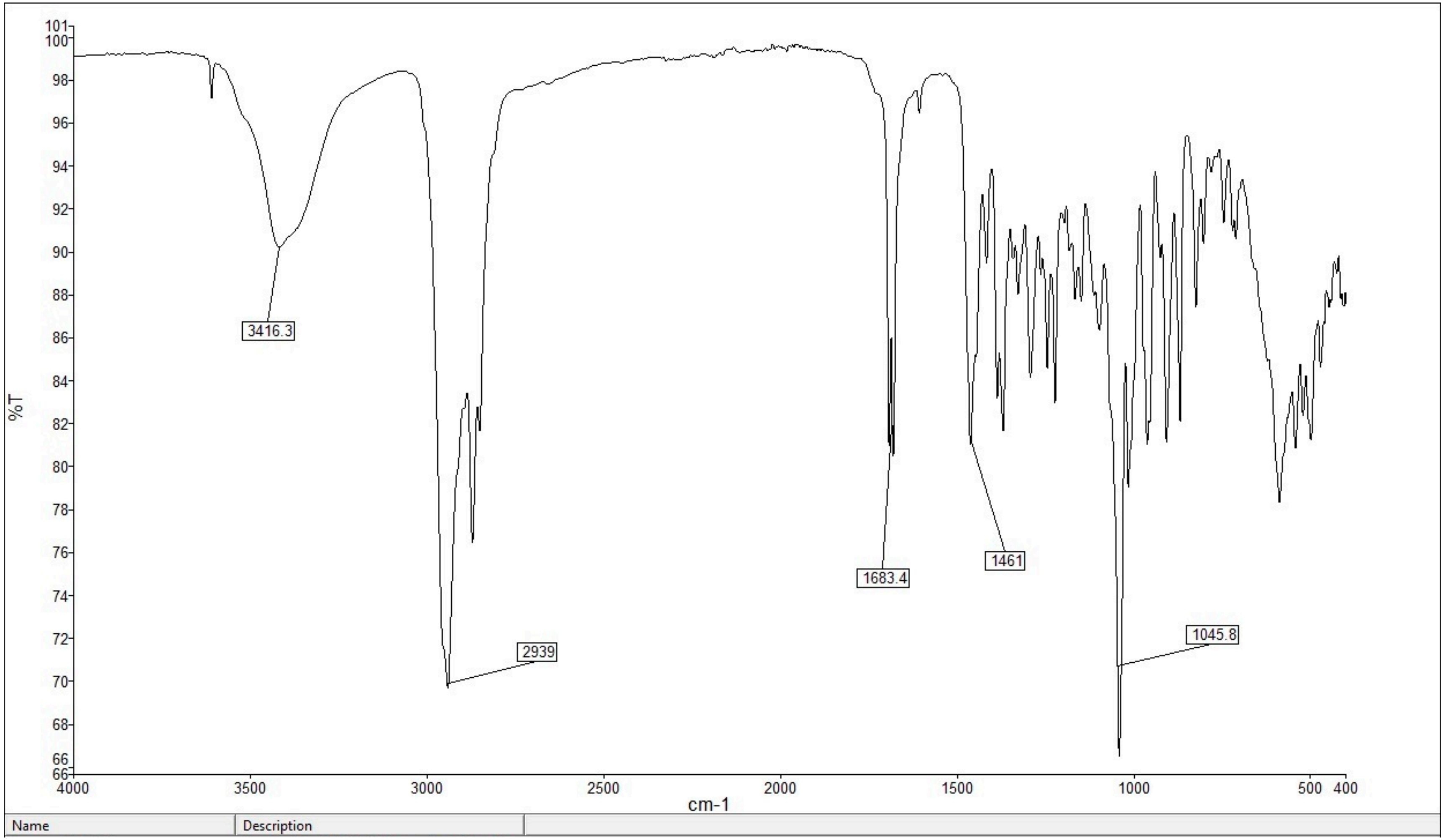

Figure S20: FT-IR spectrum of stigmasterol (**5**)

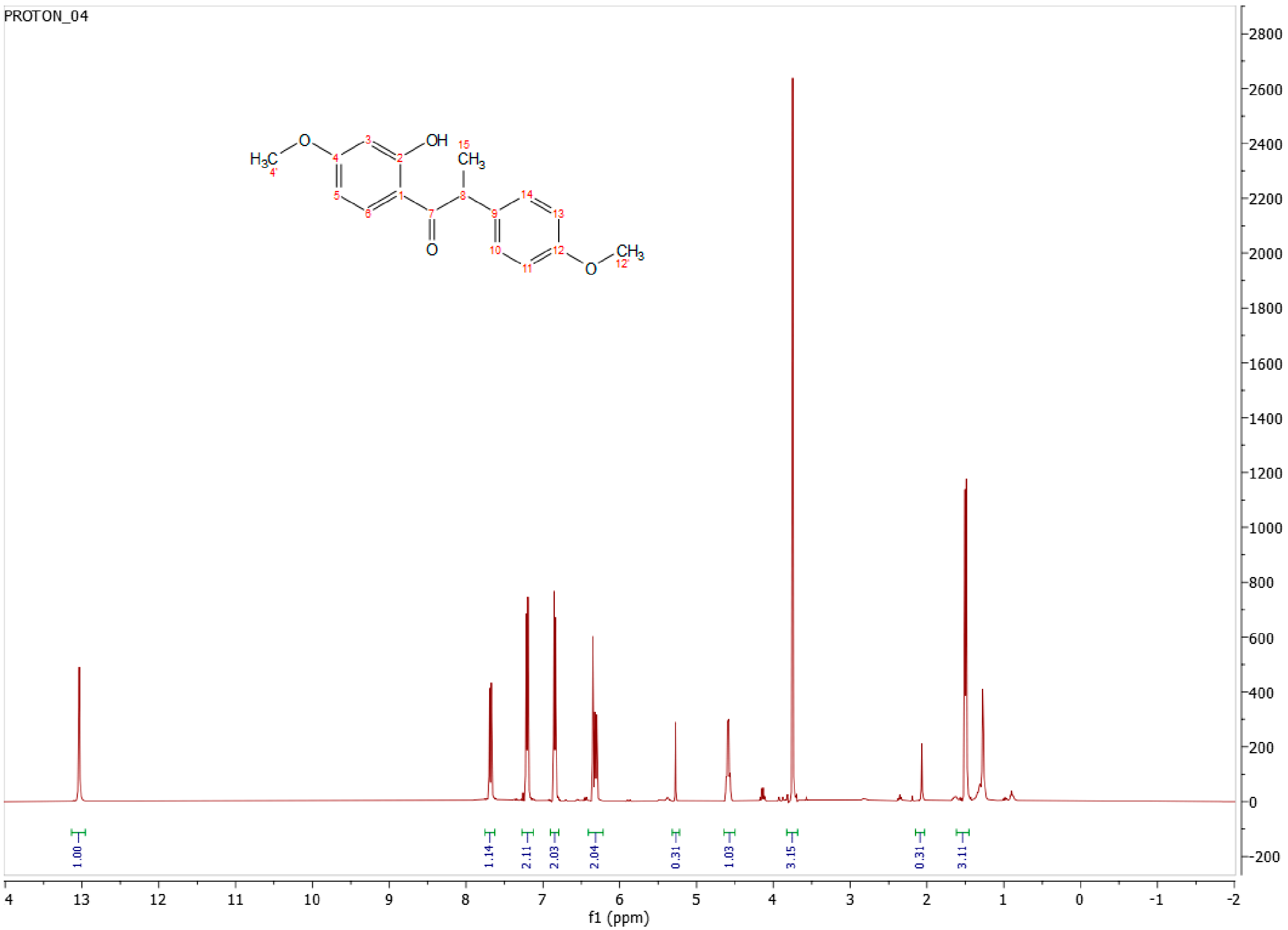

Figure S21: ^{1}H NMR spectrum of (±) -4-*O*-Methylangolensin (**6**) recorded in $CDCl_3$.

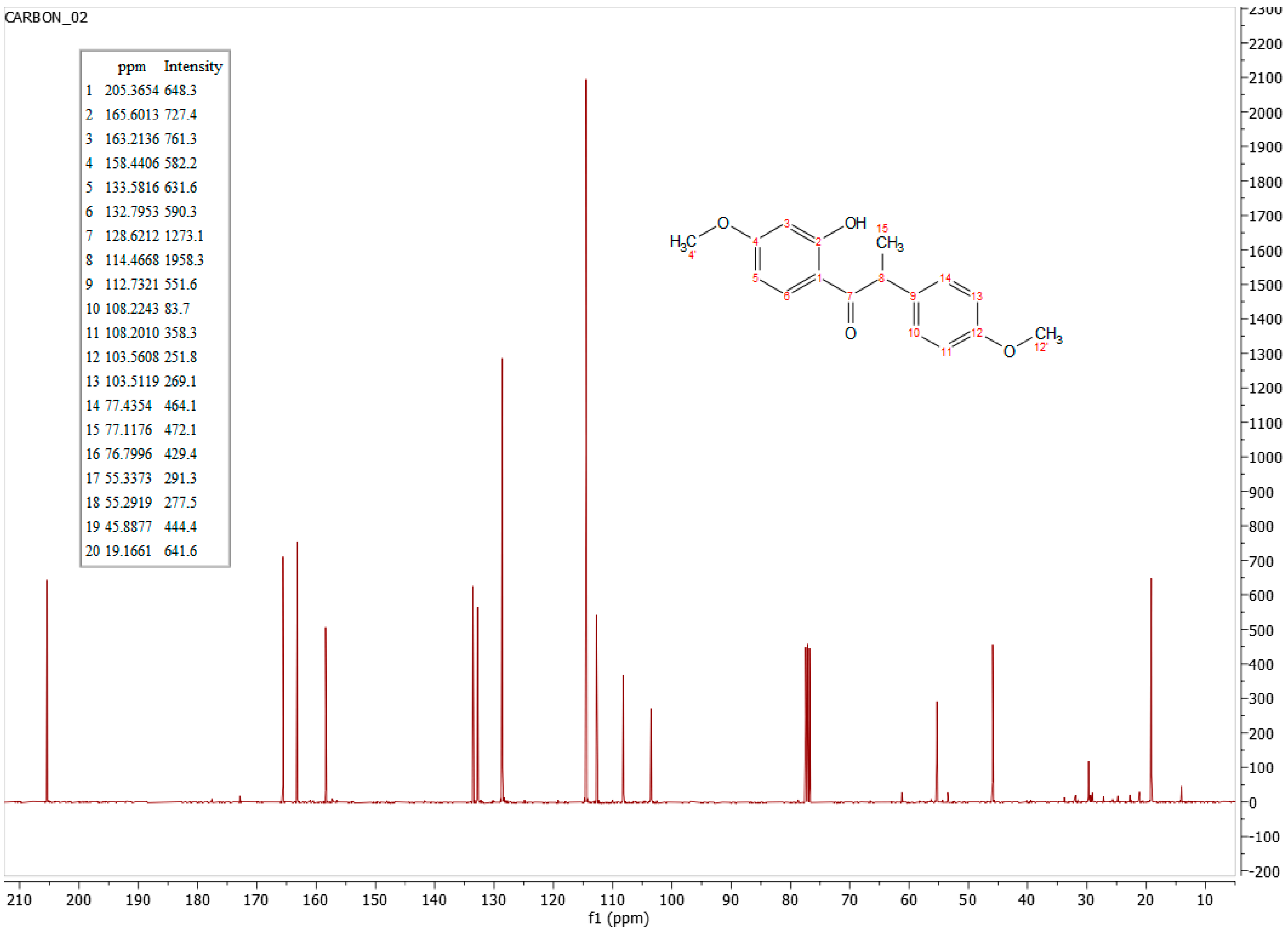

Figure S22: ^{13}C NMR spectrum of (±) -4-*O*-Methylangolensin (**6**) recorded in $CDCl_3$

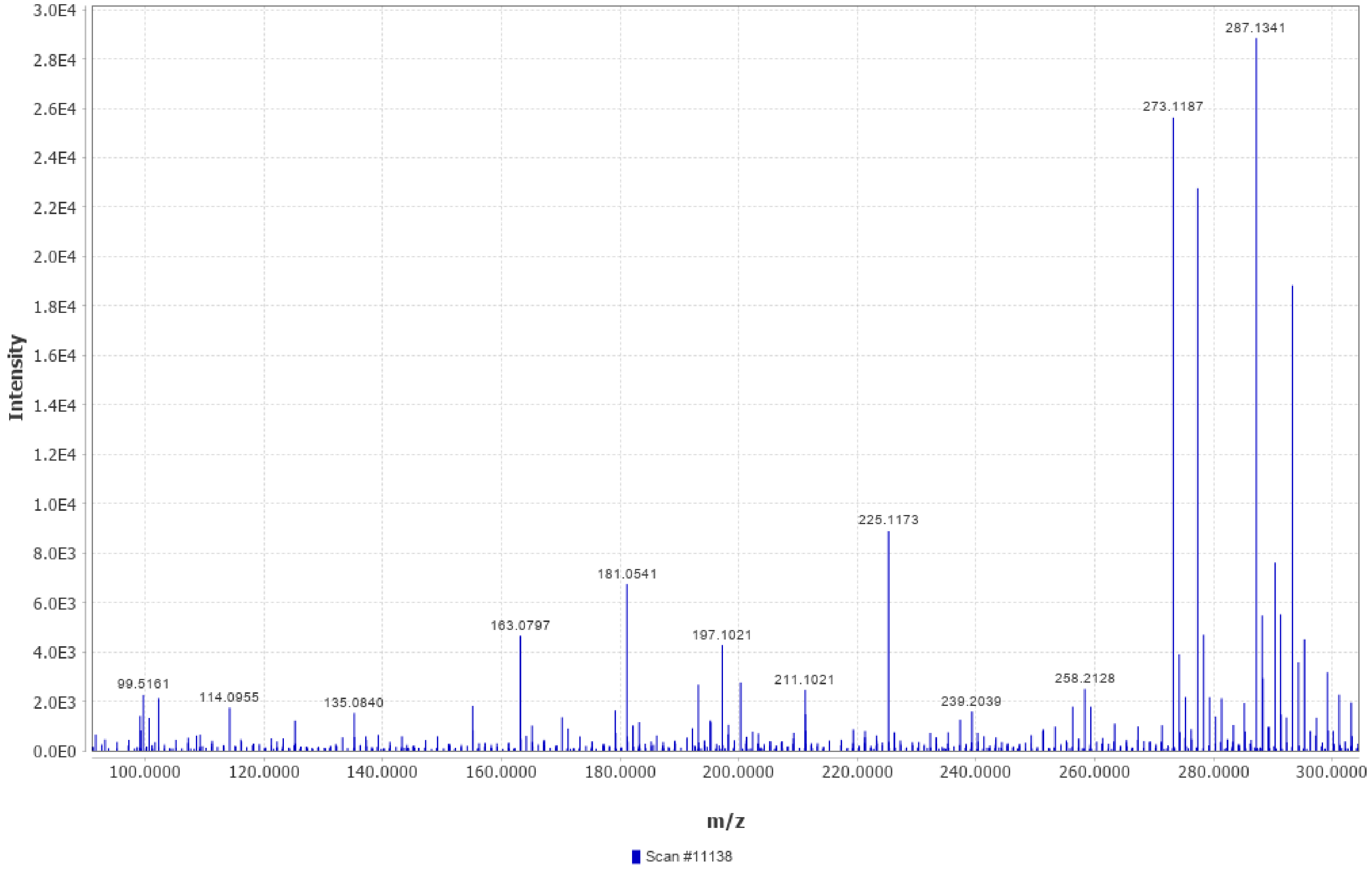

Figure S23: MS spectrum of (±) -4-*O*-Methylangolensin (**6**).

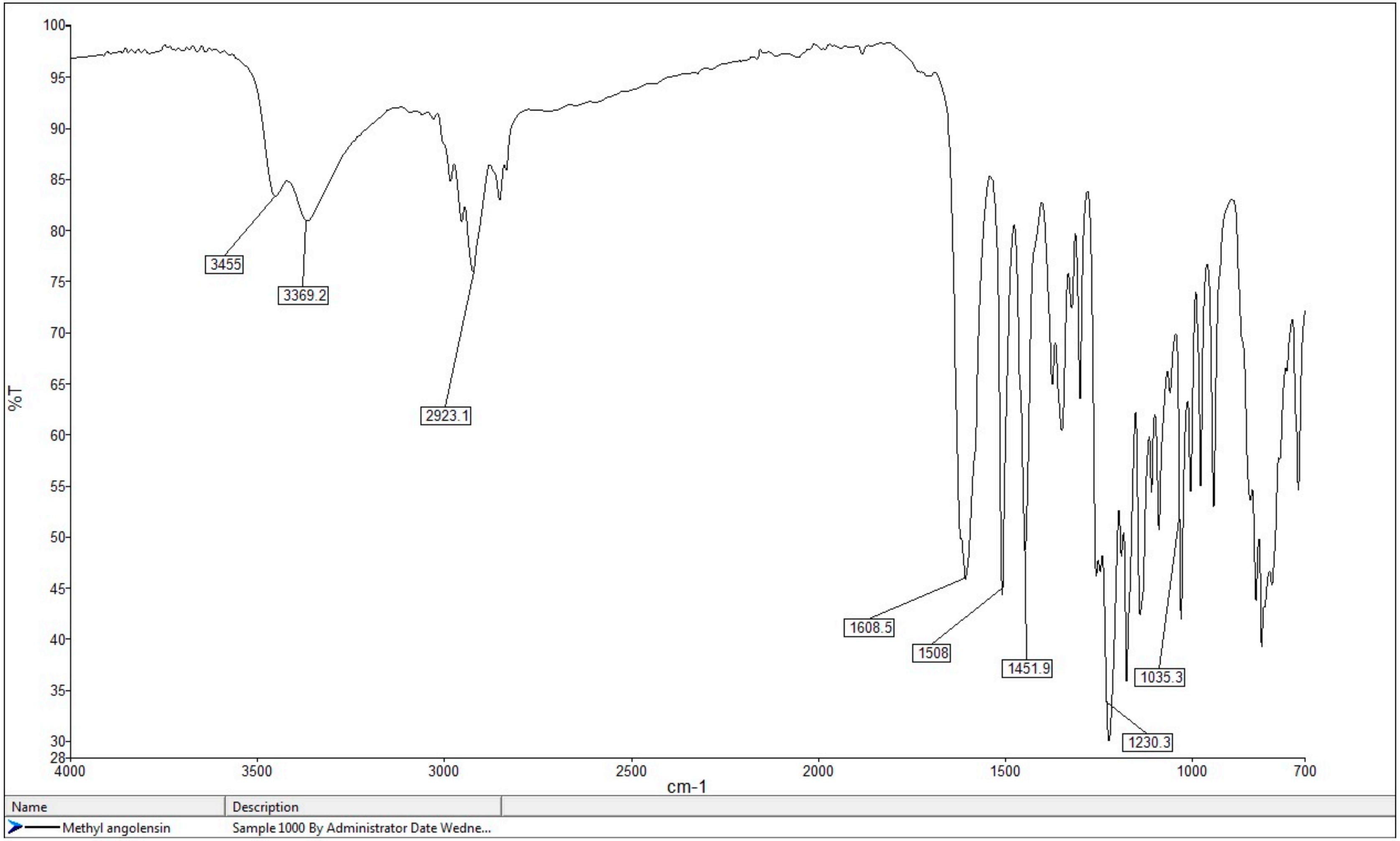

Figure S24: FT-IR spectrum of (±) -4-*O*-Methylangolensin (**7**).

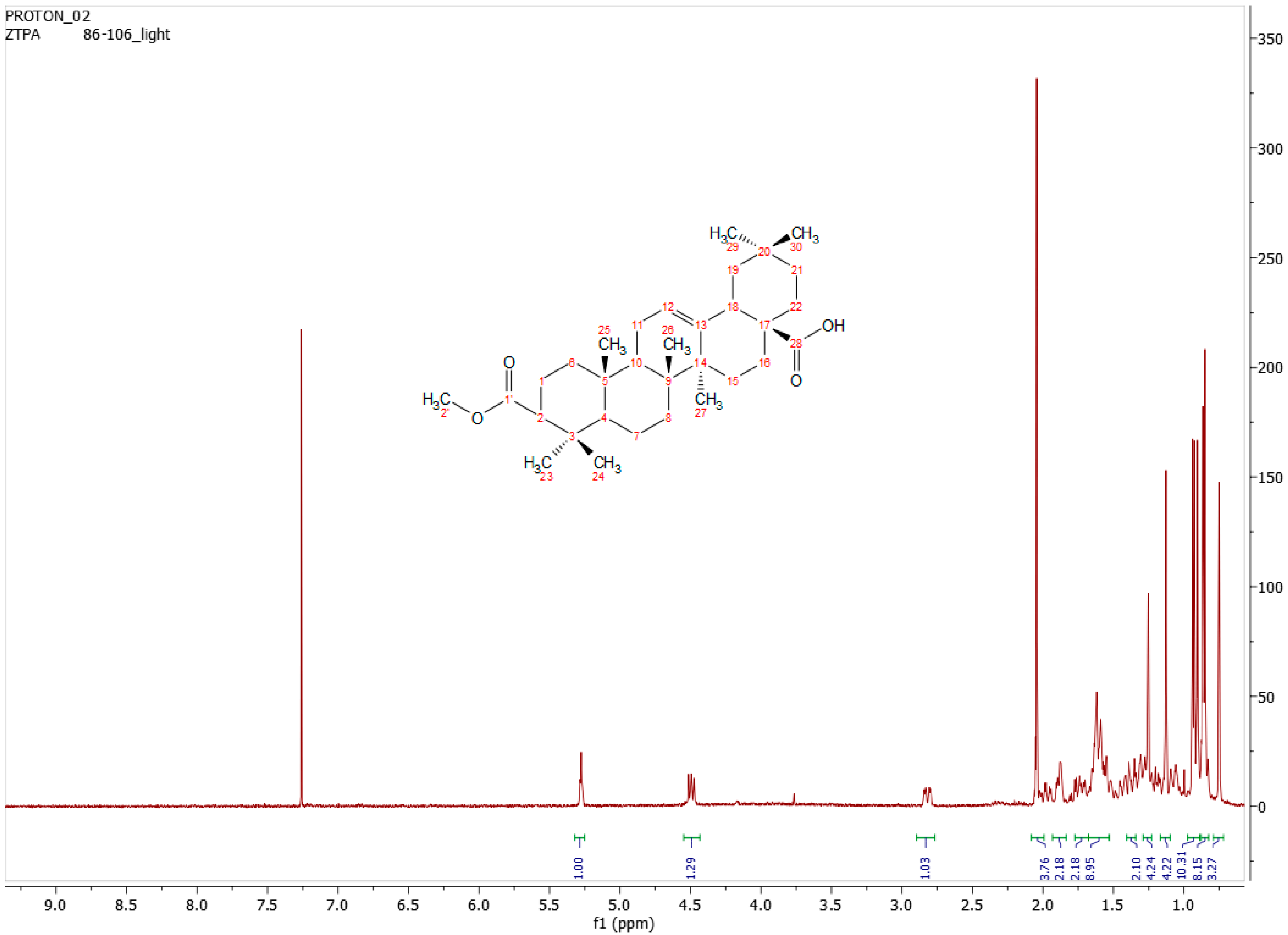

Figure S25: ^{1}H NMR spectrum of Oleanolic acid acetate (**7**) recorded in $CDCl_3$.

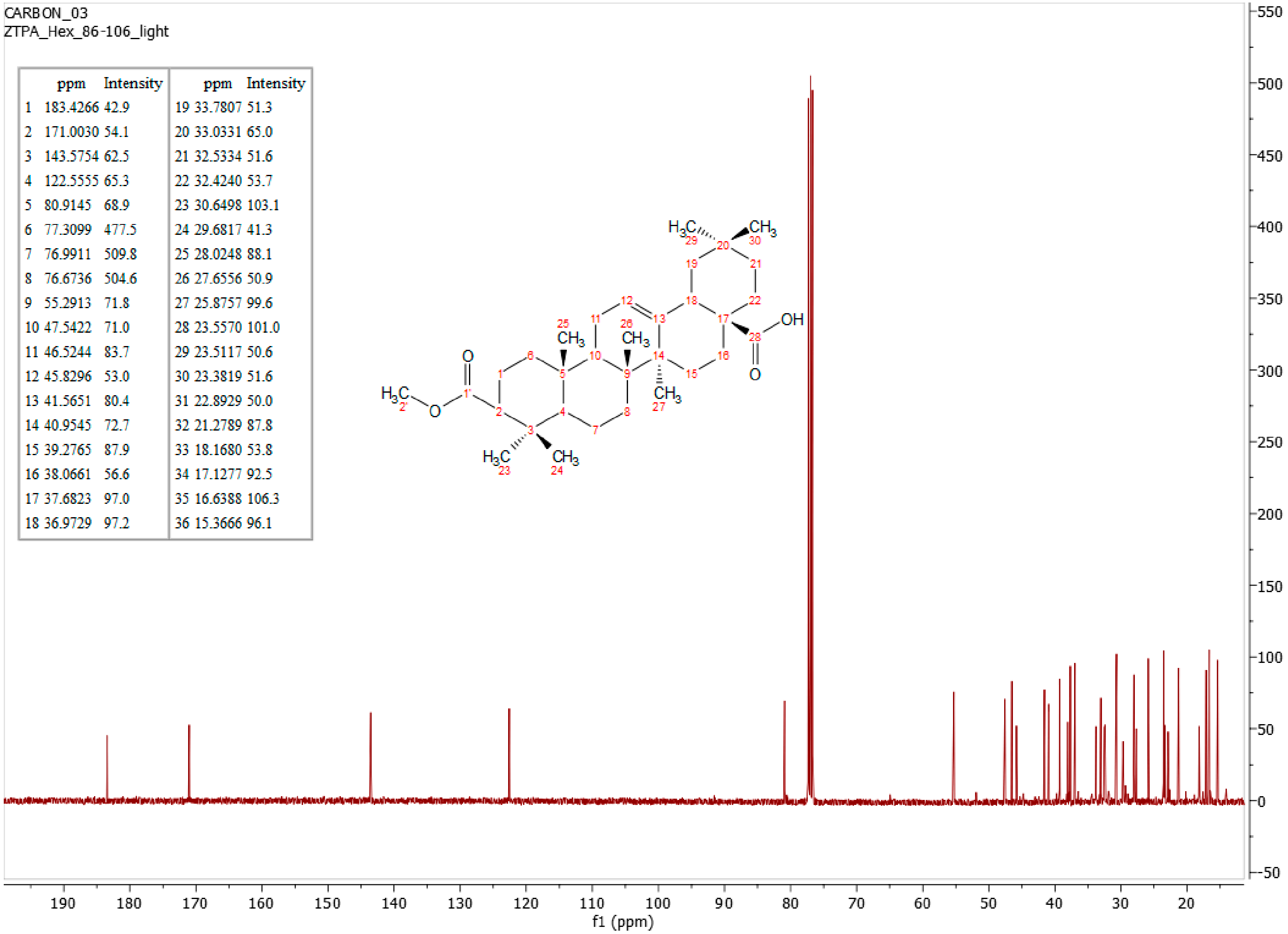

	ppm	Intensity		ppm	Intensity
1	183.4266	42.9	19	33.7807	51.3
2	171.0030	54.1	20	33.0331	65.0
3	143.5754	62.5	21	32.5334	51.6
4	122.5555	65.3	22	32.4240	53.7
5	80.9145	68.9	23	30.6498	103.1
6	77.3099	477.5	24	29.6817	41.3
7	76.9911	509.8	25	28.0248	88.1
8	76.6736	504.6	26	27.6556	50.9
9	55.2913	71.8	27	25.8757	99.6
10	47.5422	71.0	28	23.5570	101.0
11	46.5244	83.7	29	23.5117	50.6
12	45.8296	53.0	30	23.3819	51.6
13	41.5651	80.4	31	22.8929	50.0
14	40.9545	72.7	32	21.2789	87.8
15	39.2765	87.9	33	18.1680	53.8
16	38.0661	56.6	34	17.1277	92.5
17	37.6823	97.0	35	16.6388	106.3
18	36.9729	97.2	36	15.3666	96.1

Figure S26: ^{13}C NMR spectrum of Oleanolic acid acetate (**7**) recorded in $CDCl_3$

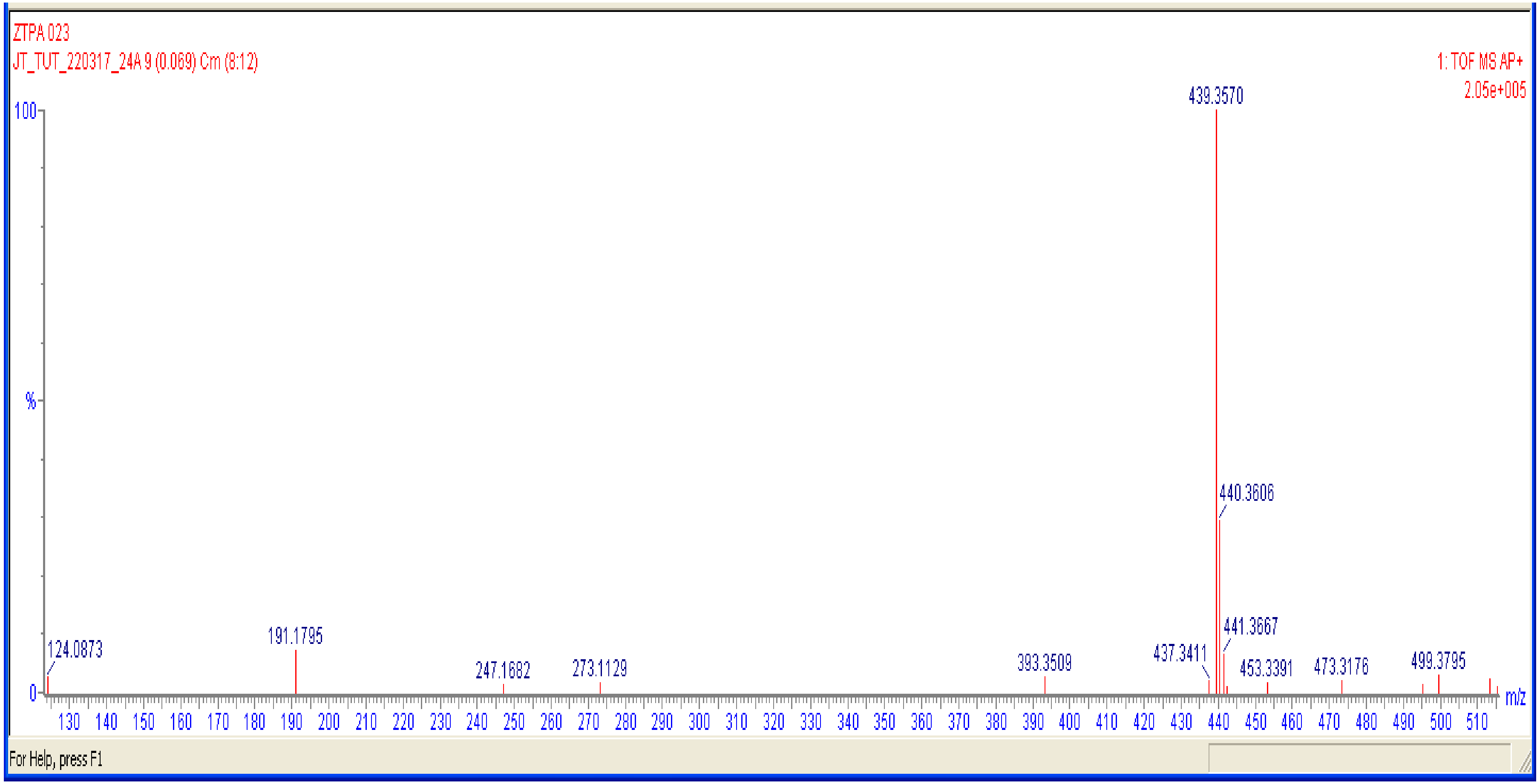

Figure S27: MS spectrum of Oleanolic acid acetate (**7**).

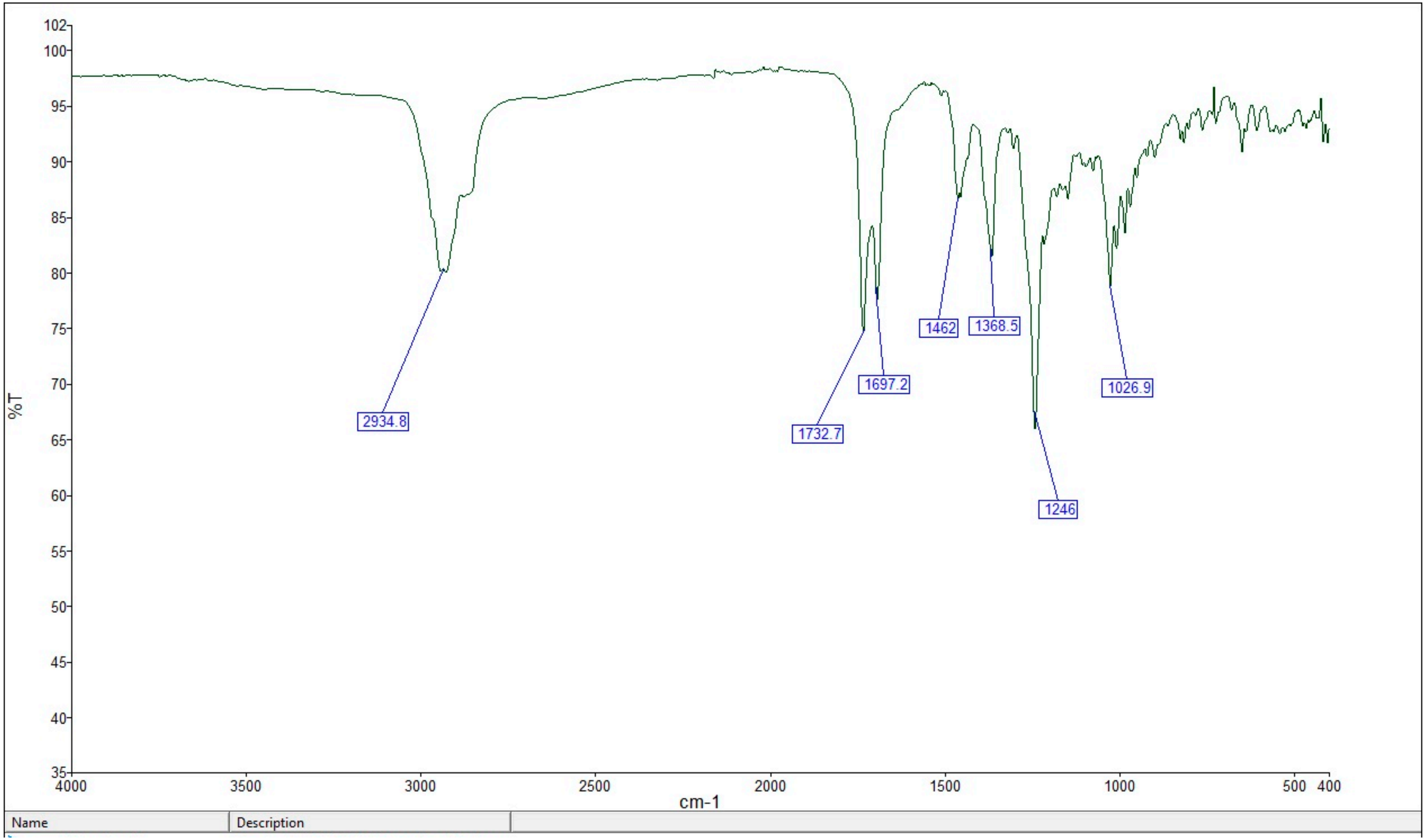

Figure S28: FT-IR spectrum of Oleanolic acid acetate (**7**).

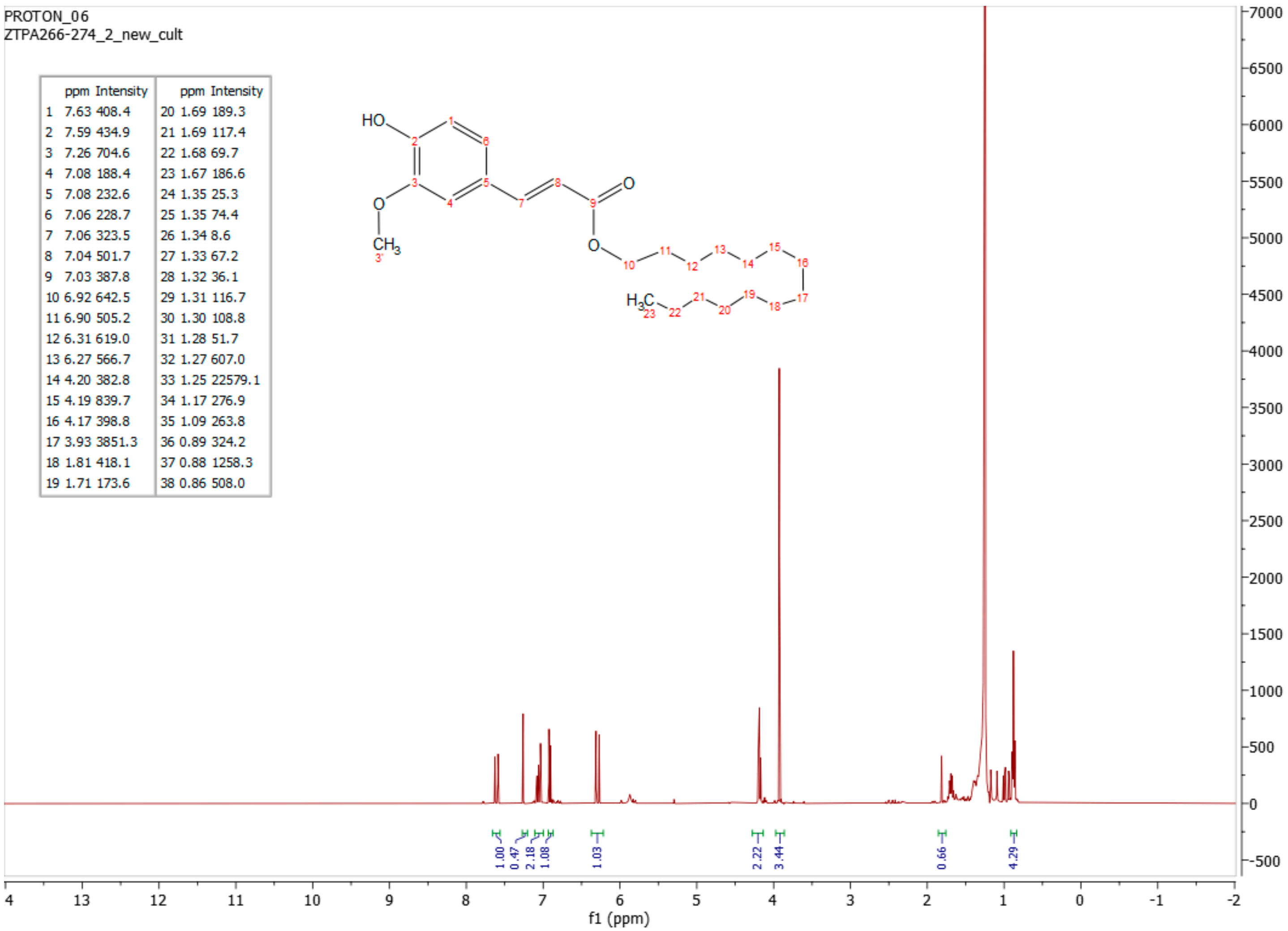

Figure S29: ^{1}H NMR spectrum of Tetradecyl (E)-ferulate (**8**) recorded in $CDCl_3$.

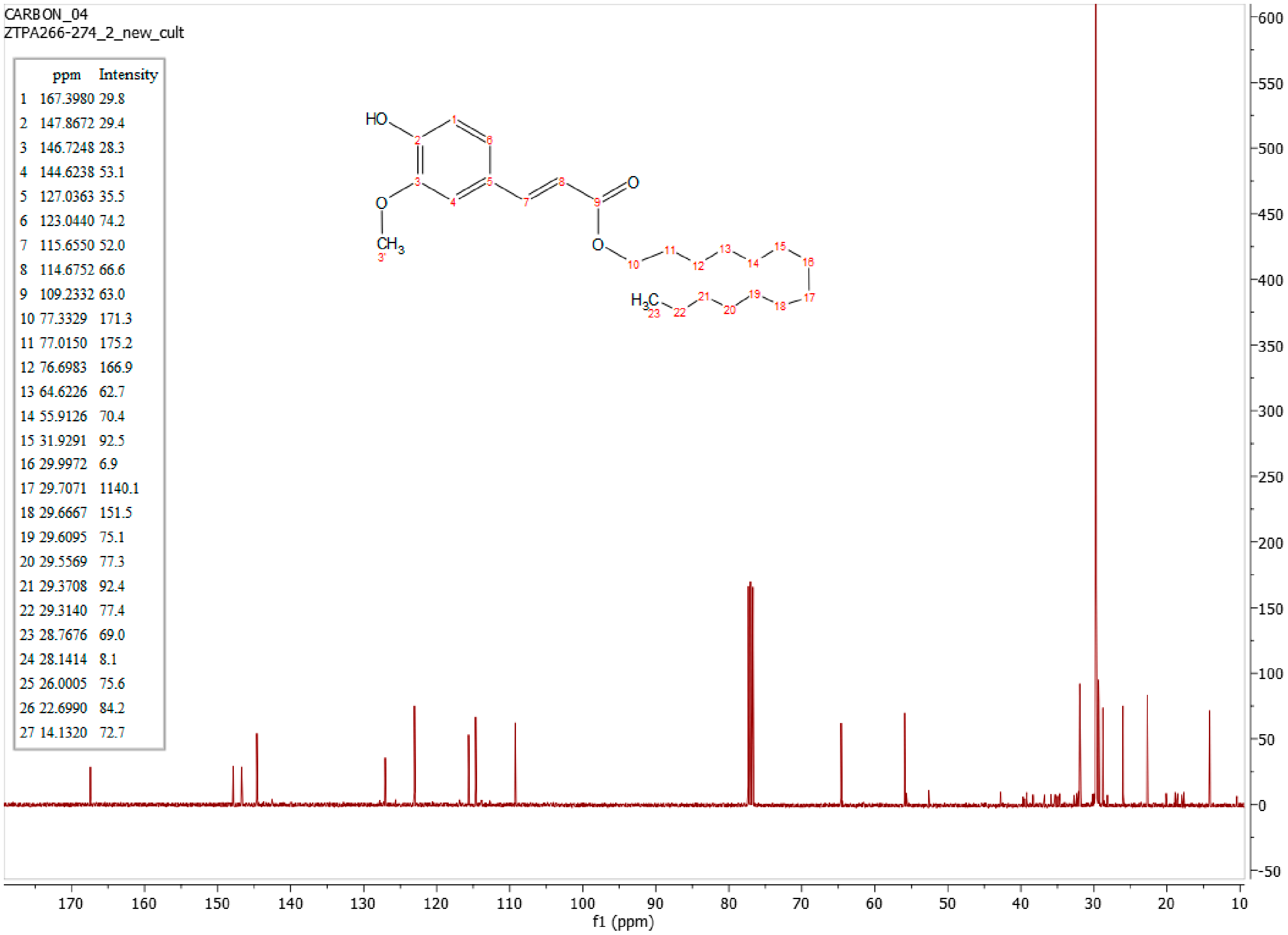

Figure S30: ^{13}C NMR spectrum of Tetradecyl (E)-ferulate (**8**) recorded in $CDCl_3$

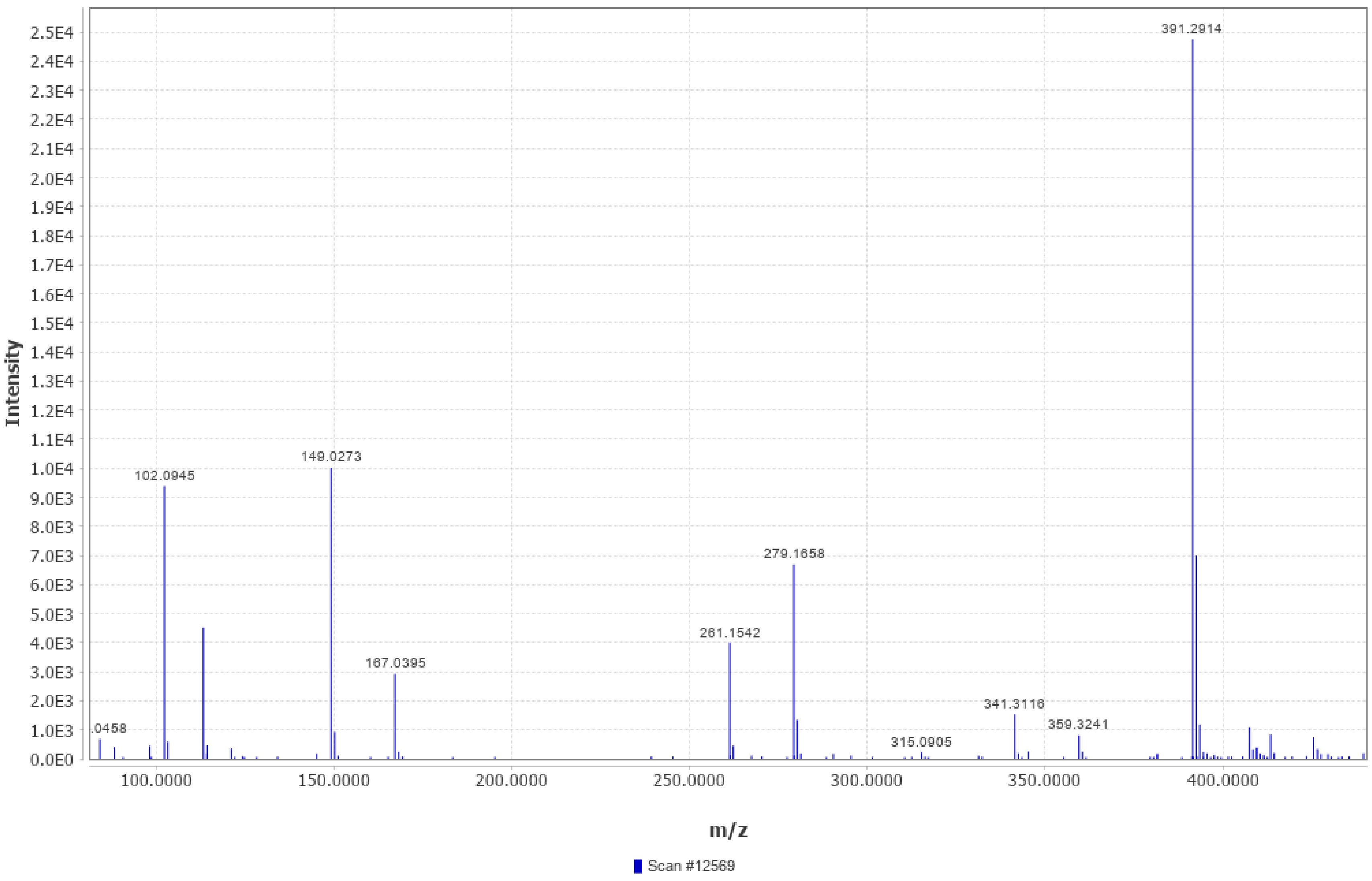

Figure S31: MS spectrum of Tetradecyl (E)-ferulate (**8**).

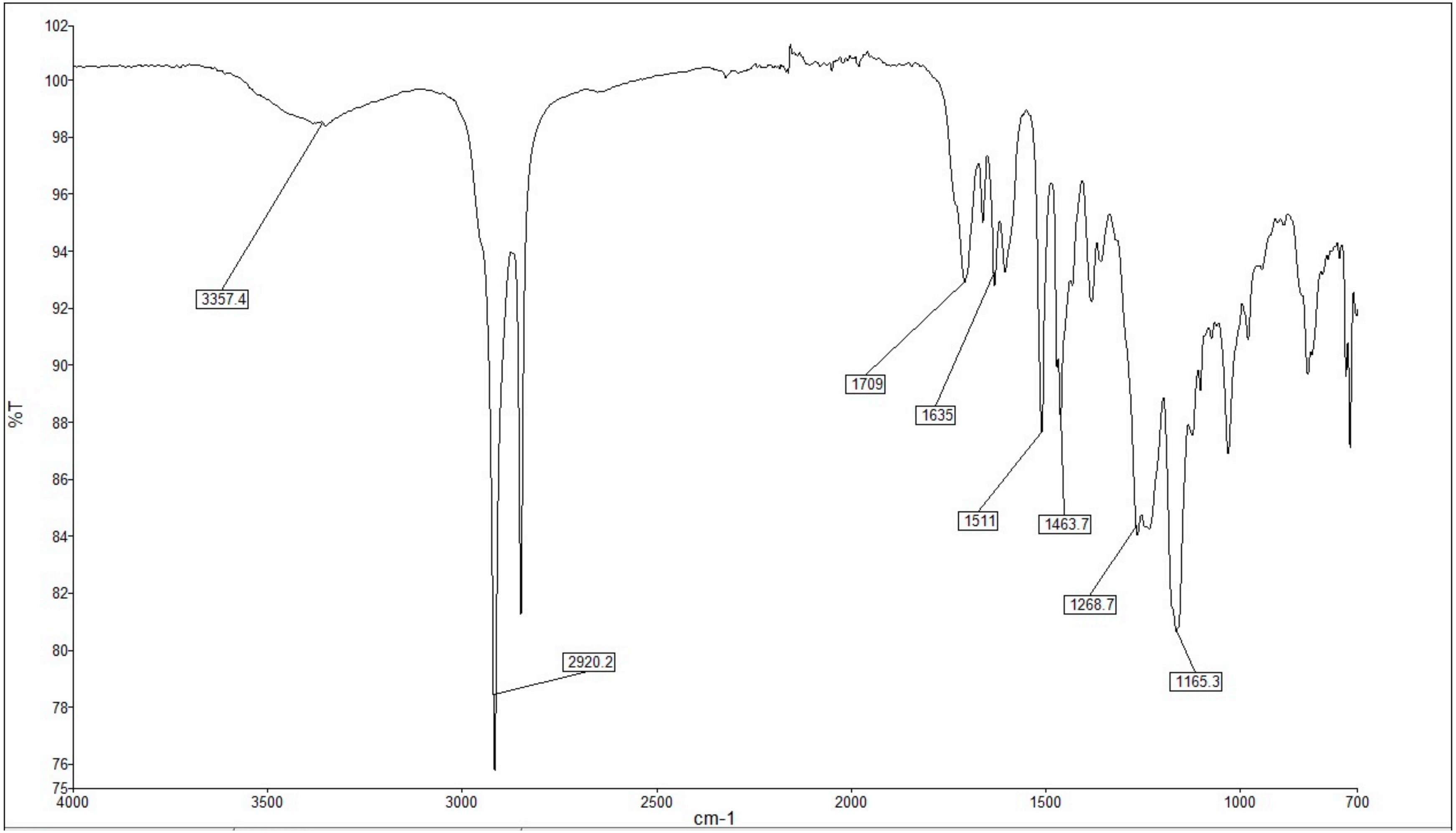

Figure S32: FT-IR spectrum of Tetradecyl (E)-ferulate (**8**).

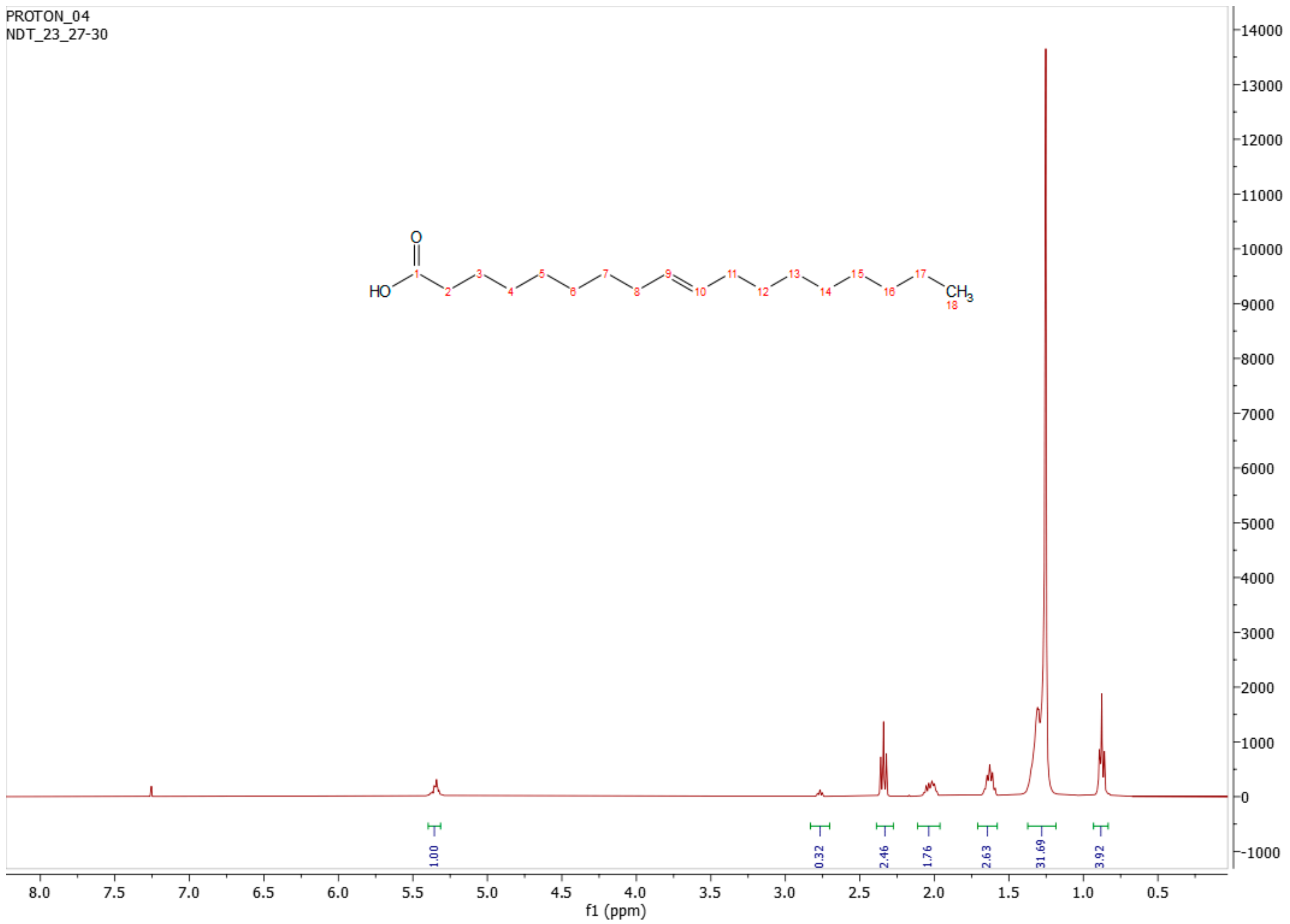

Figure S33: ^{1}H NMR spectrum of 9-Octadecenoic acid (**9**) recorded in $CDCl_3$.

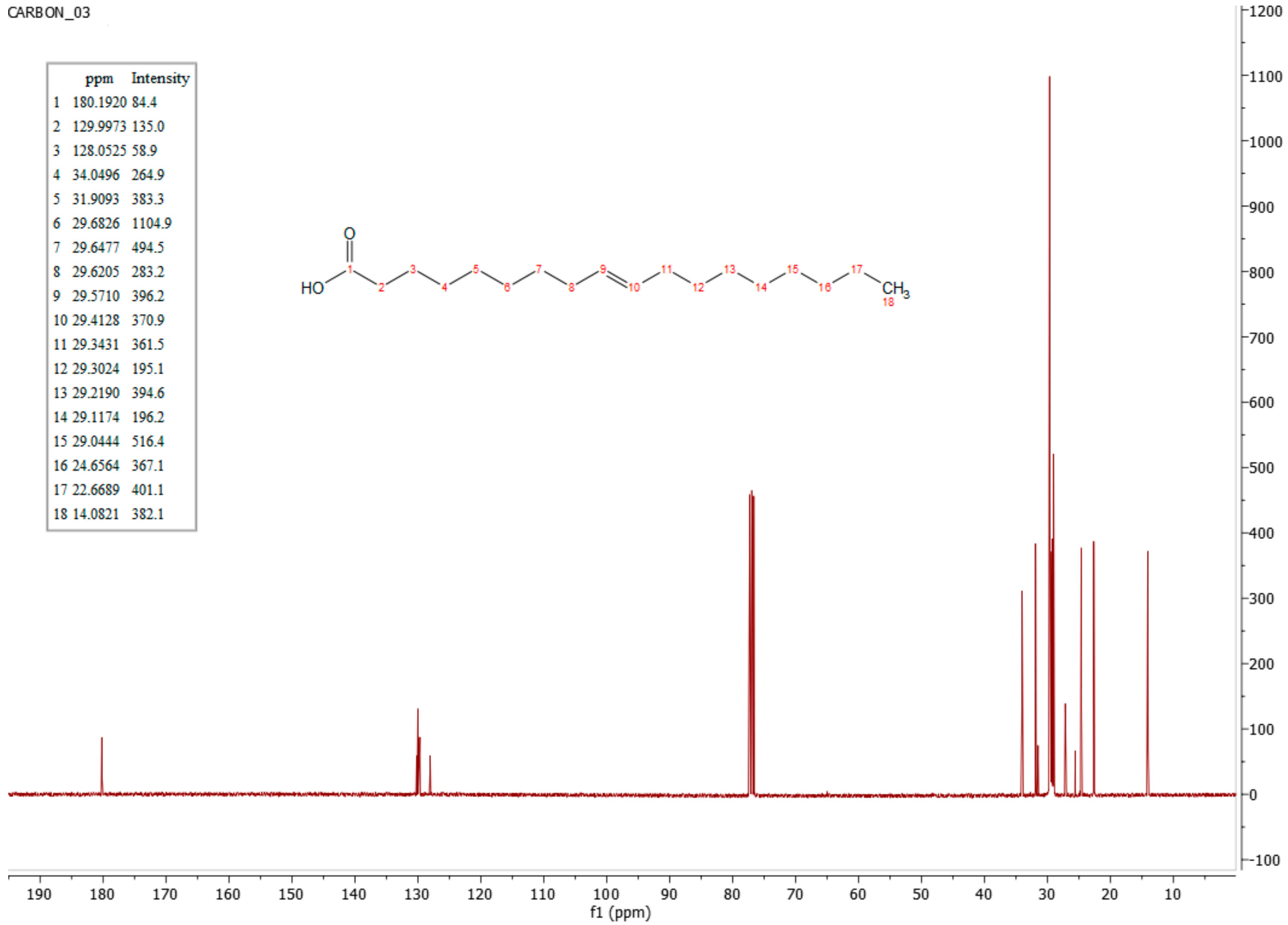

Figure S34: ^{13}C NMR spectrum of 9-Octadecenoic acid (**9**) recorded in $CDCl_3$

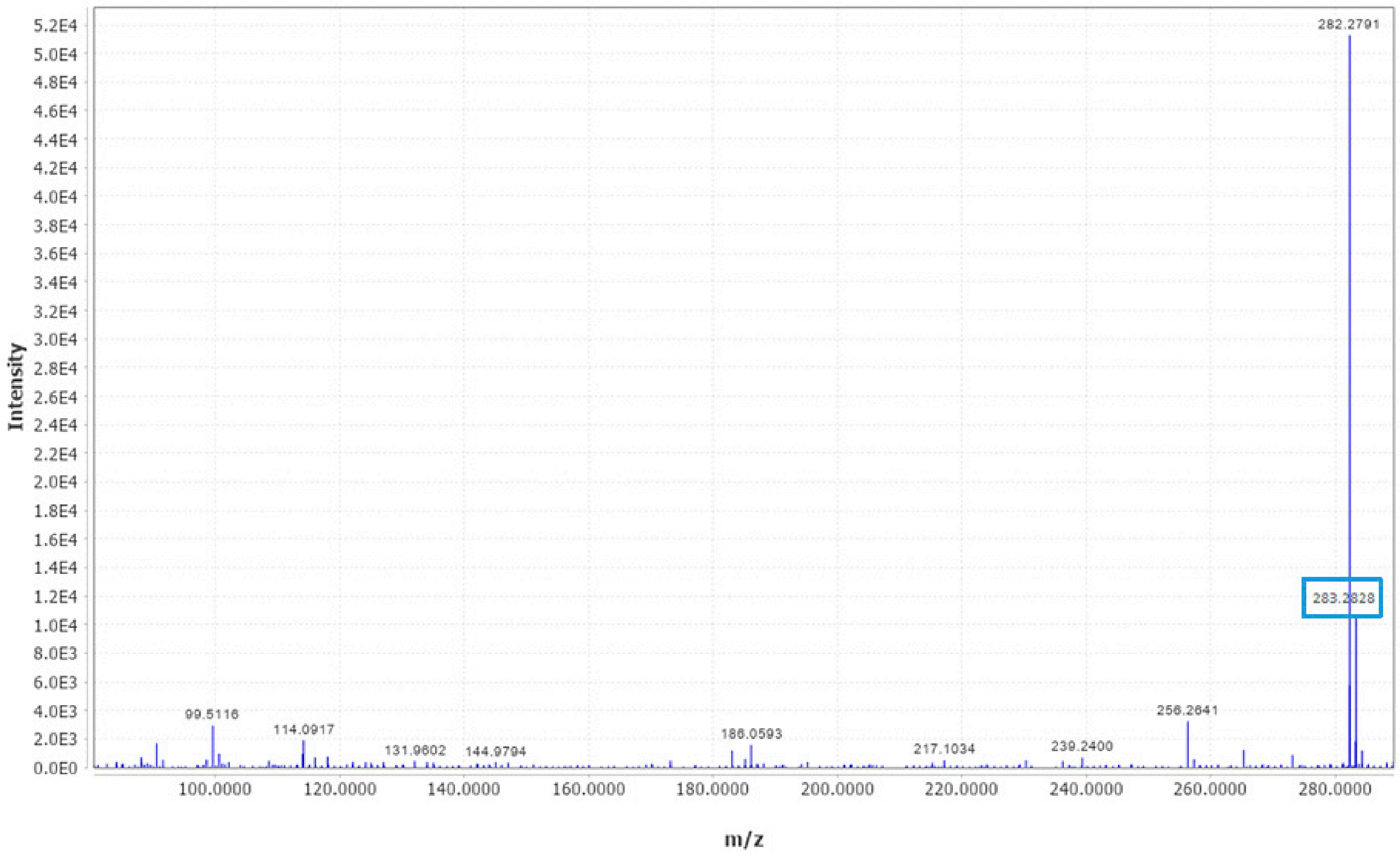

Figure S35: MS spectrum of 9-Octadecenoic acid (**9**).

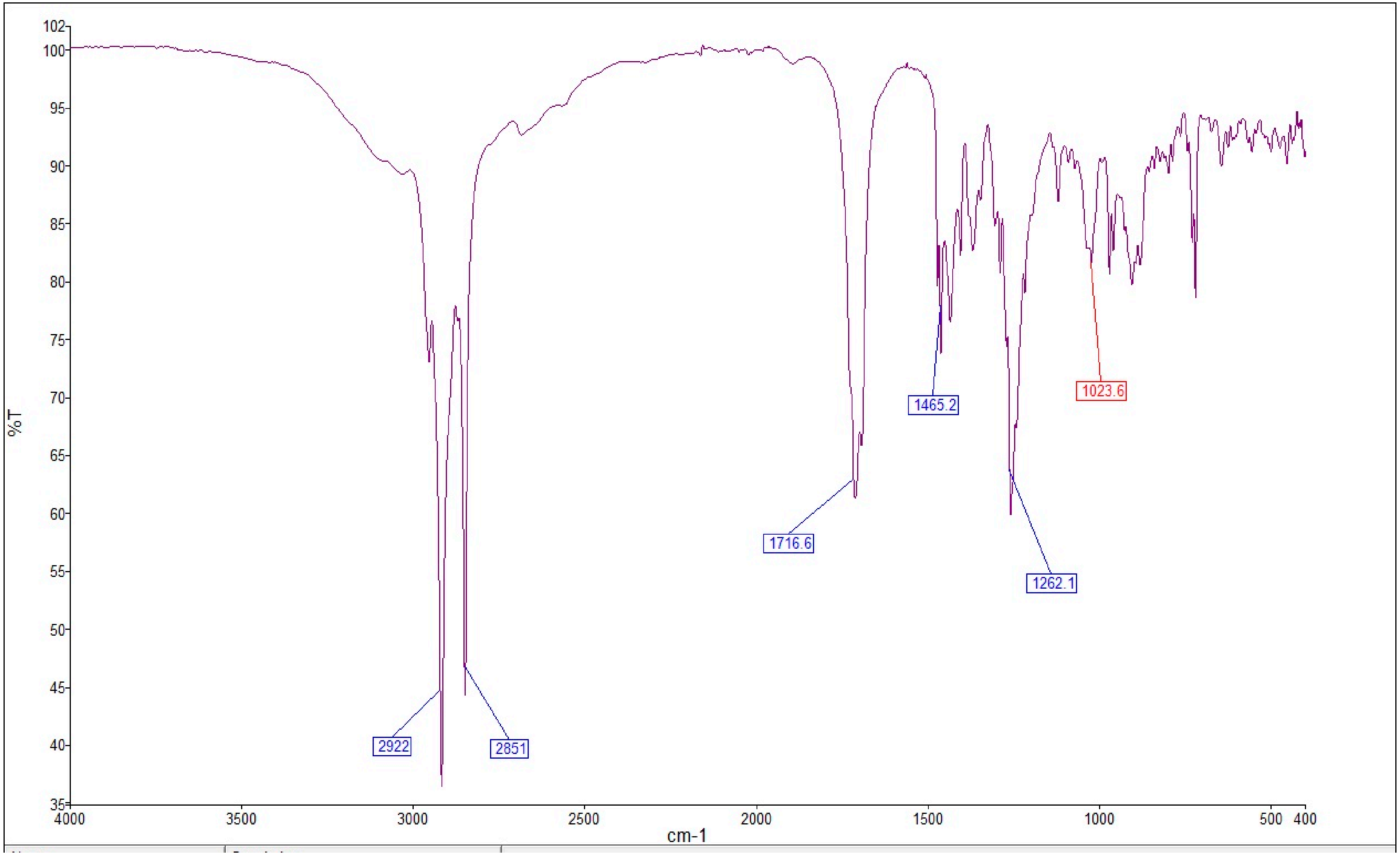

Figure S36: FT-IR spectrum of 9-Octadecenoic acid (**9**).

Table S1. ^{13}C NMR (100 MHz) data (δ value) of isolated compounds (1-9) in $CHCl_3$

C. No	13C NMR (100 MHz) δ value								
	Compound 1	Compound 2	Compound 3	Compound 4	Compound 5	Compound 6	Compound 7	Compound 8	Compound 9
1.	22.3 (CH_2)	36.1 (CH_2)	32.1 (CH_2)	38.7 (CH_2)	37.2 (CH_2)	113.0 (C)	38.1 (CH_2)	127.0 (C)	180.1 (C)
2.	41.6 (CH_2)	211.9 (C)	195.0 (C)	27.4 (CH_2)	31.7 (CH_2)	165.9 (CH)	27.7 (CH_2)	123.1 (CH)	34.2 (CH_2)
3.	213.1 (C)	76.9 (CH_2)	142.6 (CH)	79.0 (CH)	71.8 (CH)	103.6 (CH)	80.9 (CH)	114.7 (CH)	24.7 (CH_2)
4.	58.2 (CH)	54.6 (CH)	140.8 (C)	38.9 (C)	42.3 (CH_2)	55.2 (OCH_3)	37.7 (C)	147.9(C)	28.9 (CH_2)
5.	42.1 (C)	38.1 (C)	39.7 (C)	55.3 (CH)	140.8 (C)	162.4 (C)	55.3 (CH)	146.7 (C)	29.3 (CH_2)
6.	41.6 (CH_2)	40.6 (CH_2)	38.4 (CH_2)	18.3 (CH_2)	121.7 (CH)	107.7 (CH)	18.2 (CH_2)	109.3 (CH)	29.7 (CH_2)
7.	18.3 (CH_2)	17.6 (CH_2)	18.0 (CH_2)	34.3 (CH_2)	31.9 (CH_2)	132.7 (CH)	32.5 (CH_2)	144.8 (CH)	29.4 (CH_2)
8.	53.1 (CH)	53.9 (CH)	52.6 (CH)	40.8 (C)	31.9 (CH)	205.0 (C)	39.3 (C)	115.7 (CH)	27.2 (CH_2)
9.	37.4 (C)	37.6 (C)	36.7 (C)	50.4 (CH)	50.1 (CH)	46.0 (CH)	47.5 (CH)	167.4 (C)	130.0 (C)
10.	59.5 (CH)	60.4 (CH)	55.7 (CH)	37.1 (C)	36.5 (C)	19.2 (CH_3)	37.0 (C)	64 (CH_2)	128.1 (C)
11.	35.6 (CH_2)	35.0 (CH_2)	34.7 (CH_2)	20.9 (CH_2)	21.1 (CH)	133.5 (C)	23.4 (CH_2)	28.9 (CH_2)	25.7 (CH_2)
12.	30.5 (CH_2)	30.1 (CH)	30.2 (CH_2)	25.1 (CH_2)	39.7 (CH_2)	128.6 (CH)	122.6 (CH)	26.1 (CH_2)	29.2 (CH_2)
13.	39.7 (C)	39.6 (C)	39.5 (C)	38.0 (CH)	42.2	114.4 (CH)	143.6 (C)	29.3 (CH_2)	29.3 (CH_2)
14.	38.3 (C)	38.3 (C)	38.2 (C)	42.8 (C)	56.8 (CH)	55.2(OCH_3)	41.6 (C)	29.4 (CH_2)	29.6 (CH_2)
15.	32.4 (CH_2)	32.2 (CH_2)	32.2 (CH_2)	27.4 (CH_2)	24.4 (CH_2)	158.7 (C)	27.7 (CH_2)	29.5 (CH_2)	29.7 (CH_2)
16.	36.0 (CH_2)	35.8 (CH_2)	35.9 (CH_2)	35.6 (CH_2)	29.7 (CH_2)	114.4 (CH)	22.9 (CH_2)	29.5 (CH_2)	31.9 (CH_2)
17.	30.0 (C)	30.0 (C)	30.0 (C)	43.0 (C)	56.8 (CH)	128.6 (CH)	46.5 (C)	29.6 (CH_2)	22.7 (CH_2)
18.	42.8 (CH)	42.7 (CH)	42.6 (CH)	48.3 (CH)	12.0 (CH_3)	-	41.0 (CH_2)	29.6 (CH_2)	14.1 (CH_3)
19.	35.3 (CH)	35.3 (CH_2)	35.3 (CH_2)	48.0 (CH)	19.4 (CH_3)	-	45.8 (CH_2)	29.7 (CH_2)	-
20.	28.1 (C)	28.1 (CH_2)	28.1 (C)	151.0 (C)	40.5 (CH)	-	30.7 (C)	29.7 (CH_2)	-
21.	32.8 (CH_2)	32.8 (CH_2)	32.7 (CH_2)	29.8 (CH_2)	21.2 (CH_3)	-	33.8 (CH_2)	29.8 (CH_2)	-
22.	39.2 (CH_2)	39.2 (CH_2)	39.3 (CH_2)	40.0 (CH_2)	138.3 (CH)	-	32.4 (CH_2)	22.7 (CH_2)	-
23.	6.8 (CH_3)	10.8 (CH_3)	10.4 (CH_3)	28.0 (CH_3)	129.3 (CH)	-	16.6 (CH_3)	14.2 (CH_3)	-

24.	14.6 (CH_3)	14.2 (CH_3)	17.7 (CH_3)	15.3 (CH_3)	51.2 (CH)	-	28.0 (CH_3)	55.9 (OCH_3)	-
25.	17.9 (CH_3)	17.6 (CH_3)	18.6 (CH_3)	16.1 (CH_3)	31.9 (CH)	-	15.4 (CH_3)	-	-
26.	20.2 (CH_3)	19.9 (CH_3)	18.9 (CH_3)	16.0 (CH_3)	21.1 (CH_3)	-	17.1 (CH_3)	-	-
27.	18.6 (CH_3)	18.6 (CH_3)	20.1 (CH_3)	14.5 (CH_3)	19.0 (CH_3)	-	25.9 (CH_3)	-	-
28.	32.1 (CH_3)	32.2 (CH_3)	32.1 (CH_3)	18.0 (CH_3)	25.4 (CH_2)	-	183.4 (C)	-	-
29.	35.0 (CH_3)	31.9 (CH_3)	31.8 (CH_3)	109.3 (CH_2)	12.2 (CH_3)	-	23.6 (CH_3)	-	-
30.	31.8 (CH_3)	35.3 (CH_3)	35.0 (CH_3)	19.3 (CH_3)	-	-	33.0 (CH_3)	-	-
31.	-	-	-	-	-	-	21.3 (CH_3)	-	-
32.	-	-	-	-	-	-	171.0 (C)	-	-

Table S2. ^{1}H NMR (400 MHz) data (δ value) of isolated compounds (1-5 and 7) in $CHCl_3$

C. No	^{1}H NMR (400MHz, δ, ppm)					
	Compound 1	Compound 2	Compound 3	Compound 4	Compound 5	Compound 7
1.	1.91 (1H, m)<br>1.65 (1H, m)	2.39 (1H, t, *J*=13.8 Hz)<br>2.52 (1H, dd *J*= 10.8, 2.9 Hz)	2.42 (1H, dd *J*= 17.8.0, 15.7 Hz)<br>2.53 (1H, dd *J*= 17.8, 3.64 Hz	0.91 (1H, m)	1.85 (1H, m),<br>1.08 (1H, m)	1.60, 1.05 (2H, m)
2.	2.36 (1H, m)<br>2.27 (1H, m)	-	-	1.67 (1H, m)	1.84 (1H, m), 1.50 (1H, m)	162, 188 (2H, m)
3.	-	3.81 (1H, dd *J*=11.8, 2.96 Hz)<br>3.51 (1H, d, *J*= 3.48 Hz OH-3	5.98 (1H, s, OH-3)	1.60 (1H, d, *J* = 10.0 Hz)	3.51 (tdd, *J* = 6.1, 4.4, 5.1 Hz)	4.51 (1H, t, *J*= 7.6 Hz)
4.	2.21 (1H, q, *J*=6.7 Hz)	1.29 (m)	-	1.52 (1H, m)	2.30 (1H, m)<br>2.23 (1H, m)	-
5.	-	-	-	3.19 (1H, dd, *J* =4.4, 6.4 Hz)	-	0.85 (1H, m)
6.	1.73 (1H, m)<br>1.23 (1H, m)	1.06 (1H, m)<br>1.85 (1H, br d *J*=12.8 Hz)	1.93 (1H, dd *J*= 12.0, 2.52 Hz)<br>181 (1H, m)	-	5.34 (d, *J* = 5.2 Hz)	1.52, 1.40 (2H, m)
7.	1.47 (1H, m)<br>1.38 (1H, m)	1.43 (1H, m)<br>1.48 (1H, m)	1.50 (1H, m)<br>1.44 (1H, m)	0.70 (1H, d, *J* = 5.2 Hz)	1.99(1H, m)<br>1.53 (1H, m)	1.40, 1.28 (2H, m)
8.	1.38 (1H, m)	1.29 (1H, m)	1.37 (1H, m)	1.52 (1H, m)	1.49 (1H, m)	-
9.	-	-	-	1.39 (1H, m)	0.93 (1H, m)	1.55 (1H, t)
10.	1.53 (1H, m)	1.30 (1H, m)	1.80 (1H, m)	<1.38> (2H, m)	-	-
11.	1.45 (1H, m)<br>1.27 (1H, m)	1.20 (1H, m)<br>1.30 (1H, m)	1.32 (1H, m)<br>1.26 (1H, m)	-	<1.50> (1H, m)	1.88, 1.62 (2H, dd, *J*= 6.0, 3.7 Hz)
12.	1.32 <1H, m>	1.33 (2H, m)	1.33 (2H, m)	1.26 (1H, m)	2.04 (1H, m)<br>1.17 (H, m)	5.28 (1H, t, *J*= 3.7 Hz)
13.	-	-	-	-	-	-

14.	-	-	-	1.40 (1H, m)	1.02 (1H, m)	-
15.	1.46 (1H, m)<br>1.25 (1H, m)	1.27 (1H, m)<br>1.51 (1H, m)	1.50 (1H, m)<br>1.30 (1H, m)	1.25 (1H, m)	1.56 (1H, m)<br>1.03 (1H, m)	1.05, 172 (2H, m)
16.	1.55 (1H, m)	1.33 (1H, m)<br>1.51 (1H, m)	1.54 (1H, m)<br>1.38 (1H, m)	1.67 (1H, d, *J*=9.6Hz),	1.26 (1H, m)<br>1.05 (1H, m)	1.98, 1.94 (2H, dd, *J* = 9.4, 4.0 Hz)
17.	-	-	-	1.02 (1H, m)	1.16 (1H, m)	-
18.	1.53 (1H, m)	1.52 (1H, m)	1.55 (1H, m)	1.67 (1H, t)	0.69 (3H, s)	2.84 (2H, dd, *J*= 9.4, 4.6 Hz)
19.	1.44 (1H, m)	1.16 (1H, m)<br>1.32 (1H, m)	1.20 (1H, m)	-	1.02 (3H, s)	1.62, 1.18 (2H, m)
20.	-	-	-	1.71 (1H, m)	2.04 (1H, m)	-
21.	1.46 (1H, m)<br>1.26 (1H, m)	1.47 (2H, m)	1.46 (1H, m)<br>1.28 (1H, m)	1.02 (1H, m)	1.02 (3H, s)	1.22, 1.19 (2H, m)
22.	1.45 (1H, m)<br>0.93 (1H, m)	1.46 (2H, m)	1.51 (1H, m)	1.46 (1H, m)	5.14 (1H, dd, *J* = 8.5, 6.6 Hz)	1.76, 1.57 (2H, m)
23.	0.86 (3H, d, *J*=6.3 Hz)	1.05 (3H, d, *J*=6.6 Hz)	1.80 (3H, s)	1.36 (1H, m)	5.05 (1H, dd, *J* = 8.5, 6.4 Hz)	0.85 (3H, s)
24.	0.71 (3H, s)	1.03 (3H, s)	0.93 (3H, s)	-	1.52 (1H, m)	0.86 (3H, s)
25.	0.86 (3H, s)	0.87 (3H, s)	0.98 (3H, s)	1.36 (1H, t)	1.52 (1H, m)	0.94 (3H, s)
26.	0.98 (3H, s)	1.00 (3H, s)	1.09 (3H, s)	2.38 (1H, td, *J*= 5.2,5.6 Hz)	0.83 (3H, d, *J* = 5.8 Hz)	0.75 (3H, s)
27.	1.04 (3H, s)	0. 97 (3H, s)	1.00 (3H, s)	-	0.80 (3H, d, *J* = 5.8 Hz)	1.13 (3H, s)
28.	1.16 (3H, s)	1.17 (3H, s)	1.17 (3H, s)	1.91 (1H, m)	1.42 (1H, m)<br>1.16 (1H, m)	-
29.	0.99 (3H, s)	0.98 (3H, s)	0.99 (3H, s)	1.33 (1H, m)	0.80 (3H, t, *J* = 6.4 Hz)	0.93 (3H, s)
30.	0.94 (3H, s)	0.94 (3H, s)	0.93 (3H, s)	1.38 (1H, m)	-	0.90 (3H, s)
31.	-	-	-	-	-	2.04 (3H, s)

Table S3. ^{1}H NMR (400 MHz) data (δ value) of isolated compounds (6, 8 and 9) in $CHCl_3$

Carbon No	^{1}H NMR (400MHz, δ, ppm)		
	Compound 6	Compound 8	Compound 9
1.	-	-	-
2.	13.05 (1H, s, -OH)	7.07 (1H, dd, *J*= 8.2, 1.6 Hz)	2.34 (2H, t, *J*= 7.5 Hz)
3.	6.36 (1H, d, *J*= 2.6 Hz)	6.92 (1H, d, *J*= 8.1 Hz)	1.63 (2H, m)
4.	3.76 (3H, s, OCH_3)	-	1.31 (2H, m)
5.		-	1.26 (2H, m)
6.	6.33 (1H, dd, *J*= 8.0, 2.6 Hz)	7.03 (1H, d, *J*= 2.0 Hz)	1.26 (2H, m)
7.	7.70 (1H, d, *J*= 8.0 Hz)	7.36 (1H, d, *J*= 15.9 Hz)	1.30 (2H, m)
8.	-	6.31(1H, d, *J*= 15.9 Hz)	2.02 (2H, m)
9.	4.60 (1H, q, *J*= 8.0 Hz)	-	5.35 (1H, m)
10.	1.50 (3H, s)	4.19 (2H, t, *J*= 6.7 Hz)	5.35 (1H, m)
11.	-	1.69 (2H, m)	2.78 (2H, m)
12.	7.21 (1H, d, *J*= 7.0 Hz)	1.38 (2H, m)	1.25 (2H, m)
13.	6.85 (1H, d, *J*= 7.0Hz)	1.34 (2H, m)	1.26 (2H, m)
14.	3.76 (3H, s, OCH_3)	1.33 (2H, m)	1.26 (2H, m)
15.	-	1.32 (2H, m)	1.26 (2H, m)
16.	6.83 (2H, d, *J*= 7.0 Hz)	1.31 (2H, m)	1.27 (2H, m)
17.	7.21 (2H, d, *J*= 7.0 Hz)	1.32 (2H, m)	1.30 (2H, m)
18.	-	1.30 (2H, m)	0.88 (3H, t, *J*= 7.0 Hz)
19.	-	1.26 (2H, m)	-
20.	-	1.26 (2H, m)	-
21.	-	1.25 (2H, m)	-
22.	-	1.25 (2H, m)	-
23.	-	3.91 O-CH_3 (3H, s)	-

24.	-	0.88 (3H, t, *J*= 6.6 Hz)	-
